# Differential associations of tau extent and load with brain metabolic and cognitive dysfunction in Alzheimer's disease

**DOI:** 10.1016/j.ebiom.2026.106460

**Published:** 2026-08-29

**Authors:** Arthur C. Macedo, Lydia Trudel, Seyyed Ali Hosseini, Gleb Bezgin, Kely Quispialaya-Socualaya, Joseph Therriault, Nesrine Rahmouni, Cécile Tissot, Marcel S. Woo, Christian Limberger, Luiza S. Machado, Débora G. Souza, Delphine Oliva-Lopez, Stuart Mitchell, Tevy Chan, Brandon Hall, Étienne Aumont, Stijn Servaes, Marina Pereira Gonçalves, Ana Paula Bernardes Real, Jesse Klostranec, Paolo Vitali, Karine Provost, Tharick A. Pascoal, Eduardo R. Zimmer, Jean-Paul Soucy, Pedro Rosa-Neto, Alzheimer's Disease Neuroimaging Initiative

**Affiliations:** a Translational Neuroimaging Laboratory, The McGill University Research Centre for Studies in Aging, McConnell Brain Imaging Centre, Montreal Neurological Institute, Montréal, Québec, Canada; b Douglas Hospital Research Centre - Centre intégré universitaire de santé et services sociaux de l’Ouest-de-l’Île-de-Montréal, Verdun, Quebec, Canada; c Montreal Neurological Institute, Montréal, Québec, Canada; d Lawrence Berkeley National Laboratory, 1 Cyclotron Rd, Berkeley, CA, USA; e Translational Neurodegeneration Laboratory, Department of Neurology, University Medical Center Hamburg-Eppendorf, Hamburg, Germany; f Translational Neurodegeneration Laboratory, Department of Neurology, University Hospital Bonn, Bonn, Germany; g Graduate Program of Biological Sciences: Biochemistry, Universidade Federal do Rio Grande do Sul, Porto Alegre, Brazil; h Department of Psychiatry and Neurochemistry, Institute of Neuroscience and Physiology, The Sahlgrenska Academy, University of Gothenburg, Mölndal, Sweden; i Department of Nuclear Medicine, Centre Hospitalier de l'Université de Montréal, Québec, Canada; j Department of Psychiatry, School of Medicine, University of Pittsburgh, Pittsburgh, PA, USA; k Department of Neurology, School of Medicine, University of Pittsburgh, Pittsburgh, PA, USA; l Brain Institute of Rio Grande do Sul, PUCRS, Porto Alegre, Brazil; m Hospital Moinhos de Vento, Porto Alegre, Brazil; n Departament of Pharmacology, Universidade Federal do Rio Grande do Sul, Porto Alegre, Brazil; o Graduate Program of Biological Sciences: Pharmacology and Therapeutics, Universidade Federal do Rio Grande do Sul, Porto Alegre, Brazil; p The Peter O'Donnell Jr. Brain Institute (OBI), University of Texas Southwestern Medical Centre (UTSW), Dallas, TX, USA

**Keywords:** Alzheimer disease, Spatial extent of tauopathy, Neurofibrillary tangles, Positron-emission tomography, Neurodegeneration, Glucose metabolism

## Abstract

**Background:**

Reduced [^18^F]Fluorodeoxyglucose ([^18^F]FDG)-PET uptake is a core imaging feature of Alzheimer's disease (AD). While tau load correlates with this metabolic signature, it remains unclear whether the spatial extent of tauopathy (SEOT) more accurately explains brain glucose hypometabolic patterns. Here, we compared SEOT versus tau load to determine their ability to predict brain hypometabolic signatures in AD.

**Methods:**

We performed a cross-sectional study of amyloid-β positive participants from ADNI (n = 150) and an atypical AD subset from the McGill University Research Centre for Studies in Ageing (MCSA; n = 44). Participants underwent [^18^F]AV1451 or [^18^F]MK6240 tau-PET and [^18^F]FDG-PET. Tau load was indexed with regional SUVR, and SEOT with the proportion of abnormal voxels. Linear regressions related temporal and whole-cortex tau-PET load or SEOT to [^18^F]FDG-PET. We also compared the accuracy of tau-PET metrics for identifying AD-like hypometabolism. Spearman correlations assessed SEOT/tau load-FDG associations at regional and network levels. Partial Least Squares (PLS) regression investigated whether distributed tau load and SEOT predicted [^18^F]FDG-PET signatures. Structural equation modelling and hierarchical linear models assessed associations between tau metrics and cognition dependent and independent of [^18^F]FDG-PET.

**Findings:**

Whole-cortex SEOT best predicted decreased signal in the [^18^F]FDG-PET AD-meta-ROI. SEOT also performed better in classifying AD-related brain hypometabolism. Across regions and networks, SEOT performed similarly or better than tau load in predicting metabolic dysfunction. Voxelwise analyses suggested complementary predictive value of SEOT and tau load, each capturing slightly distinct spatial associations with [^18^F]FDG-PET. PLS demonstrated partially non-redundant contributions from tau load and SEOT. Cortical SEOT showed the strongest predictive value for cognition.

**Interpretation:**

SEOT provides complementary, independent, and often stronger predictive value than tau load for brain metabolism, particularly for network-level dysfunction. SEOT may improve diagnostic characterisation and prediction of cognitive impairment beyond [^18^F]FDG-PET.

**Funding:**

TRIAD is supported by the Weston Brain Institute, Canadian Institutes of Health Research, Canadian Consortium of Neurodegeneration and Aging, Brain Canada Foundation, the Fonds de Recherche du Québec – Santé, and the Colin J Adair Charitable Foundation. ADNI is funded by the National Institute on Aging, the National Institute of Biomedical Imaging and Bioengineering, and the Canadian Institutes of Health Research.

**Research in context**

**Evidence before this study**

We searched PubMed on January 19, 2026, for studies examining the relationship between tau neurofibrillary tangle pathology and neurodegeneration in Alzheimer's disease (AD), using the terms “Alzheimer disease”, “tau”, and “neurodegeneration”. Prior work has consistently shown that tau pathology is more closely related to neurodegeneration than amyloid pathology and that tau-PET provides meaningful predictive value for neurodegenerative changes in AD. However, despite growing interest in disentangling different dimensions of tau pathology, the literature examining the differential associations of tau spatial extent versus tau load with markers of neurodegeneration remains limited.

**Added value of this study**

This study extends previous evidence linking tau spatial extent to cognitive outcomes in AD by demonstrating that tau extent better tracks distributed brain metabolic dysfunction than conventional tau load metrics. By directly comparing tau spatial extent and tau load in their associations with brain hypometabolism across multiple analytical levels and methodological approaches, our results suggest a distinct and potentially complementary role for these tau-PET metrics in capturing tau-related neurodegenerative processes.

**Implications of all the available evidence**

Disentangling the respective contributions of tau spatial extent and tau load to neurodegeneration may help guide metric selection in clinical trials and research studies and improve mechanistic understanding of how tau pathology drives neurodegenerative change. Our findings also support the potential utility of extent-based tau metrics in both research and clinical settings, as they provide a regionally unbiased, cortex-wide assessment that may be particularly informative for identifying atypical or non-canonical tau patterns relevant to diagnosis and disease characterisation.

## Introduction

Tau pathology plays a central role in the progression of Alzheimer's disease (AD) and is closely associated with neurodegeneration.^1^^,^^2^ Tau neurofibrillary tangles (NFT) burden is often assessed with positron emission tomography (PET) imaging, where the standardised uptake value ratio (SUVR) is the most widely used metric to quantify their load within predefined brain regions.^1^, ^2^, ^3^ While regional SUVR is a valuable marker for disease staging and diagnosis,^4^^,^^5^ it may provide an incomplete picture of the spatial distribution of tau pathology as it is generally assessed using averaged uptake.^6^ Because neuronal dysfunction often reflects not only local tau burden but also the broader spatial distribution of pathology,^7^ there is increasing interest in image-derived metrics that move beyond tracer intensity alone.

The spatial extent of tauopathy (SEOT) is a PET-based metric to quantify the proportion of tau-positive voxels across the cortex,^8^, ^9^, ^10^ thereby delineating the cortical footprint of tau spread.^8^, ^9^, ^10^ By capturing a spatial dimension of tau pathology distinct from conventional tau load measures, SEOT may differentially associate with downstream events such as neurodegeneration indexed by [^18^F]Fluorodeoxyglucose PET ([^18^F]FDG-PET).^2^ Disentangling these relationships may clarify how distinct aspects of tau pathology relate to tissue dysfunction and guide outcome selection in trials targeting tau propagation versus local accumulation. We therefore compared whether SEOT and tau load differentially associate with regional patterns of [^18^F]FDG-PET hypometabolism and related cognitive dysfunction in AD. As a secondary aim, we evaluated the ability of SEOT and tau load to discriminate AD from non-AD metabolic patterns in an independent cohort of patients with suspected atypical AD who were referred for [^18^F]FDG-PET as part of their clinical evaluation.

## Methods

### Study design and participants

#### Alzheimer's Disease Neuroimaging Initiative (ADNI)

For the primary analyses of this cross-sectional study, we selected participants from the ADNI cohort^11^ recruited across North America. Eligibility criteria required that participants be amyloid-β (Aβ) positive and undergo tau-PET and [^18^F]FDG-PET imaging at the same time point. We included cognitively unimpaired (CU) individuals and participants diagnosed with mild cognitive impairment (MCI) or AD dementia following standard criteria.^11^ Details on ADNI inclusion and exclusion criteria are available elsewhere.^11^

#### McGill University Research Centre for Studies in Aging (MCSA)

To accomplish our secondary aim, we selected individuals who met criteria for undergoing [^18^F]FDG-PET as part of their diagnostic workup. Participants were recruited from the MCSA, a memory clinic located in Montreal, Canada.^12^ We included patients with cognitive impairment who (1) developed cognitive symptoms at age 65 years or younger, regardless of clinical presentation, or (2) were older than 65 years and presented with predominantly non-amnestic cognitive syndromes, including non-amnestic MCI and dementia associated with atypical AD phenotypes, defined according to established diagnostic criteria.^13^, ^14^, ^15^, ^16^, ^17^ This sample reflects a clinical scenario where [^18^F]FDG-PET is indicated because the underlying pathological process remains uncertain despite standard clinical, imaging, and specialist evaluations, consistent with Canadian consensus recommendations.^18^ Patients with a diagnosis of typical probable AD^19^ were excluded because [^18^F]FDG-PET was not clinically indicated.^18^^,^^20^ To specifically evaluate the relationship between tau-PET and neurodegeneration in AD, we only included Aβ+ individuals.

### Procedures

#### ADNI

Participants underwent clinical evaluations, including the Montreal Cognitive Assessment (MoCA),^21^ and multimodal imaging with MRI, [^18^F]Flortaucipir tau-PET, and [^18^F]FDG-PET. Aβ status was determined using either [^18^F]Florbetapir Aβ-PET or cerebrospinal fluid (CSF) Aβ_42_/_40_ ratio, depending on data availability. Participants were classified as Aβ+ if they demonstrated global Aβ-PET SUVR > 1.1^22^ and/or CSF Aβ_42_/_40_ < 0.061, using the Elecsys immunoassay (http://adni.loni.usc.edu/data-samples/clinical-data/).

#### MCSA

Participants underwent evaluation by dementia specialists and neuropsychological testing. Eligible participants were invited to join the Translational Biomarkers in Aging and Dementia (TRIAD) cohort study,^5^ which included additional biomarker assessments such as [^18^F]MK6240 tau-PET imaging and Aβ status determination via [^18^F]AZD4694 Aβ-PET (Aβ+ if neocortical SUVR > 1.55^23^) and/or Lumipulse CSF Aβ_42_/_40_ (Aβ+ if ratio < 0.068^24^). In both cohorts, sex was recorded at screening based on participant self-report and was available as a binary variable (female or male).

### Neuroimaging acquisition and processing

ADNI pre-processed images were downloaded; detailed acquisition and preprocessing procedures are available elsewhere (http://adni.loni.usc.edu/datasamples/pet/). For MCSA participants, MRI was acquired using a 3T Siemens Magnetom system, and tau- and Aβ-PET imaging with a Siemens High-Resolution Research Tomograph.^25^^,^^26^ PET images underwent standard reconstruction and correction procedures.^25^^,^^26^ MCSA participants underwent [^18^F]FDG-PET brain imaging with standard commercial PET/CT scanners at the Centre Hospitalier de l'Université de Montréal.^12^ Each session consisted of a 20-min PET acquisition, followed by a low-dose CT scan for attenuation correction.^27^

PET scans from both cohorts were processed using previously described methods,^25^^,^^26^^,^^28^including registration to subject-specific T1-weighted MRI, normalisation to Montreal Neurological Institute (MNI) space, and spatial smoothing to 8 mm full-width at half-maximum. Tau- and Aβ-PET SUVR were generated using inferior and full cerebellar grey matter reference regions, respectively,^23^^,^^29^ whereas [^18^F]FDG-PET SUVR in ADNI used the pons as reference.^28^^,^^30^

### Outcomes

#### Tau-PET

To quantify tau load, we computed average SUVR within regions of interest (ROIs). SEOT was calculated as the proportion of abnormal voxels within each ROI.^6^^,^^9^^,^^31^ In ADNI, abnormal voxels were defined as those with SUVR >1.4. This threshold optimally distinguished Aβ− CU from Aβ+ MCI/AD ADNI participants and was derived using ROC analyses of voxelwise tau-PET SUVR values within a cortical MNI mask.^6^ This fixed threshold was applied across the entire tau-PET image to generate binary abnormality maps from which regional SEOT values were derived.^9^

To determine the cutoff for the MCSA dataset, we replicated this analytical framework^6^ using an independent TRIAD dataset including 138 Aβ− CU and 105 Aβ+ MCI/AD participants. ROC analyses were performed across voxel-level tau-PET SUVR thresholds ranging from 1.0 to 3.0 (Supplementary Figures S1A and B; Supplementary Table S1). Candidate thresholds were initially evaluated according to their AUC. The stability of threshold selection was then assessed using 1000 bootstrap resamples of the derivation cohort, in which ROC analyses were repeated across all candidate thresholds and the threshold yielding the highest AUC was recorded for each bootstrap sample. Thresholds were subsequently ranked according to the frequency with which they were selected as optimal across bootstrap iterations. Based on their consistently high discriminative performance and selection stability, the three highest-performing thresholds (SUVR = 1.6, 1.7, and 1.9) were retained to derive SEOT.

Sensitivity analyses used voxelwise SEOT derivation based on z-scored tau-PET images relative to the 40 youngest Aβ- CU participants in each cohort^8^^,^^10^ (Supplementary Table S2), applying thresholds of 2, 2.5, and 3 SD above the reference-group mean. Tau load and SEOT were derived from a temporal meta-ROI^5^^,^^32^ and a whole-cortex ROI.^33^

#### [^18^F]FDG-PET

In ADNI, AD-related hypometabolism was indexed using a validated [^18^F]FDG-PET meta-ROI^30^^,^^34^ comprising the bilateral angular gyri, posterior cingulate cortex, and left middle and inferior temporal gyri. An SUVR <1.21 defined [^18^F]FDG-PET hypometabolism.^30^^,^^34^^,^^35^ To assess associations between tau pathology and hypometabolism, we extracted SEOT, tau load, and [^18^F]FDG-PET SUVR from cortical regions defined by the Desikan-Killiany-Tourville (DKT) atlas^36^ and from functional networks previously implicated in AD phenotypes. We employed templates corresponding to the default mode network (DMN), language, visuospatial, salience, and frontoparietal executive control (FPEC) networks, derived from the CONN toolbox based on Human Connectome Project data.^37^ A limbic network mask was generated using the Schaefer atlas.^38^

In the MCSA cohort, board-certified nuclear medicine physicians visually assessed [^18^F]FDG-PET brain scans and corresponding 3D-stereotactic surface projection maps,^12^^,^^39^ blinded to other PET results but with access to relevant clinical information. Interpretation followed European Association of Nuclear Medicine and the European Academy of Neurology guidelines.^27^ Although all participants were Aβ+, [^18^F]FDG-PET was performed because glucose metabolism and Aβ status are not fully congruent^12^^,^^40^ and provide complementary phenotypic information.^41^

Based on cerebral hypometabolic patterns, patients were classified as AD-associated or non-AD-associated.^12^ The AD-associated group included typical hypometabolism in the posterior portions of the cingulate gyri, parietal cortices, and/or lateral temporal lobes, and patterns consistent with atypical AD variants.^12^ The non-AD-associated group included non-AD neurodegenerative patterns or no evidence of neurodegeneration.^12^^,^^42^

### Ethics

The MCSA component was approved by the MNI PET Working Committee and the Research Ethics Boards of the Douglas Mental Health University Institute and McGill University (IUSMD 16–60). The ADNI protocol was approved by the Institutional Review Board or Research Ethics Board at each participating site, with approval reference numbers being site-specific. All participants provided written informed consent, and all study procedures were conducted in accordance with the 2024 Declaration of Helsinki.

### Statistics

Statistical analyses were conducted in R (version 4.1.1). Linear regressions adjusted for age and sex examined the associations of tau load or extent with [^18^F]FDG-PET meta-ROI signal. Models were compared using Bayesian Information Criterion (BIC), F-statistics, and adjusted R^2^. ΔBIC values of 2–6, 6–10, and >10 indicated positive, strong, and very strong evidence against the higher-BIC model, respectively.^43^ Sensitivity analyses included additional adjustment for Aβ-PET burden or CSF Aβ_42_/_40_ ratio in available subsets, and separately for years of education, APOEε4 status, and clinical diagnosis in the full sample.

To assess the accuracy of tau-PET metrics for identifying brain hypometabolism, we calculated AUC using the *pROC* R package. In MCSA, logistic regressions were adjusted for the interval between [^18^F]FDG-PET and tau-PET acquisitions. AUCs and BIC were derived from these models. Pairwise AUC comparisons were performed using DeLong's test.^44^ Competitive models including both SEOT and SUVR predictors assessed independent associations with [^18^F]FDG-PET status.

Age- and sex-adjusted Spearman correlations examined associations between tau load or extent and [^18^F]FDG-PET measures across DKT regions and functional networks. For each network, SEOT, tau load, and [^18^F]FDG-PET SUVR were extracted using network masks, and correlations between the tau metrics and [^18^F]FDG-PET were computed. We then delineated core and supporting regions for each network and, for each region, compared the correlation of local (region-level) versus network-level tau-PET metrics (SUVR and SEOT, aggregated over the full network mask) with that region's [^18^F]FDG-PET SUVR. Finally, we assessed node-wise associations by correlating SEOT and tau load with [^18^F]FDG-PET SUVR across all regions composing each network. Correlation coefficients were compared with the *cocor* R package for dependent variables.^45^ Steiger p-values were adjusted for multiple comparisons using the Benjamini-Hochberg false discovery rate; significance was set at q < 0.05. Sensitivity analysis repeated the main network-level analyses further adjusting for years of education, APOEε4 status, and clinical diagnosis.

We performed age- and sex-adjusted voxel-based linear regressions using the RMINC library in R, with [^18^F]FDG-PET SUVR as the outcome and temporal or full-cortex SEOT/SUVR as predictors. Statistical significance was assessed using random field theory correction with a threshold of p < 0.001. To examine how tau-PET metrics relate to brain hypometabolism across stages of tau propagation, we quantified significant voxels within Braak-stage masks^46^ and across the whole cortex. To assess shared and predictor-specific voxelwise associations with [^18^F]FDG-PET, we computed the spatial overlap between predictor-specific t-maps and the masks described above. To compare effect sizes, voxel-level standardised β coefficients were extracted from voxels showing significance with at least one (“additive significance”) or all (“overlapping significance”) tau-PET predictors. β coefficients were compared between predictors using paired t-tests treating each voxel as a paired observation. Finally, voxelwise analyses were repeated using paired SEOT and SUVR metrics as competitive predictors to assess independent effects.

Stage-stratified analyses were performed in ADNI for the primary [^18^F]FDG-PET meta-ROI and voxelwise analyses, grouping participants into dementia and pre-dementia (CU + MCI) subgroups because only six CU participants were available for separate analyses.

Structural equation modelling (SEM) analyses were conducted in ADNI using the *lavaan* R package^47^ to investigate direct and indirect associations of tau-PET with MoCA scores. [^18^F]FDG-PET meta-ROI SUVR was included as a mediator. Separate SEMs were constructed for each tau-PET metric (temporal/whole-cortex SUVR/SEOT), estimating standardised direct, indirect, and total effects. Competitive SEMs jointly including pairs of SEOT and SUVR metrics were further performed. Models were adjusted for age, sex, and years of education and estimated using robust maximum likelihood estimation.

Hierarchical linear regression models were performed in both cohorts. MoCA scores were modelled as a function of age, sex, education, and [^18^F]FDG-PET abnormality status, after which each tau-PET metric was added separately. MCSA models were further adjusted for time between [^18^F]FDG-PET and tau-PET acquisition. Incremental cognitive explanatory value attributable to each tau measure was quantified using ΔR^2^, partial R^2^, BIC, and analysis of variance (ANOVA) comparisons against the base model. Competitive models including both SEOT and tau load predictors were built to assess independent contributions to cognition. For consistency with the ADNI analysis, only the fixed-cutoff SEOT metric based on the 1.9 SUVR threshold was used in the MCSA models, as this threshold yielded the highest AUC among the evaluated fixed cutoffs during the independent threshold discovery phase.

No formal sample size calculation was performed. The sample size was determined by the number of eligible participants with the required biomarker data.

#### Machine learning analysis

In ADNI, we tested whether distributed cortical tau load and extent collectively predict glucose metabolism using Partial Least Squares (PLS) regression, a multivariate approach that models all cortical regions simultaneously while accounting for collinearity.^48^ For each participant, regional tau load, SEOT, and [^18^F]FDG-PET SUVR values from both hemispheres were organised into subject-wise feature matrices. Three predictive models were evaluated using tau load, SEOT, or combined tau load and SEOT as predictors of [^18^F]FDG-PET SUVR. Models adjusted for age and sex. All variables were z-score standardised before model fitting. Data were randomly divided into training (80%) and testing (20%) sets. Model optimisation was performed within the training set using 5-fold cross-validation to determine the optimal number of latent PLS components. The final optimised model was evaluated on the independent testing set. Model performance was assessed using Pearson correlation coefficients (r) between predicted and observed [^18^F]FDG-PET SUVR values and root mean squared error (RMSE). PLS component weights and loadings were examined to identify the cortical tau regions contributing most strongly to metabolic patterns.

### Role of funders

The funder of the study had no role in study design, data collection, data analysis, data interpretation, or writing of the report.

## Results

We assessed 150 participants from ADNI (mean [SD] age 75.0 [7.7], 43% females) and 44 participants from the MCSA memory clinic sample (mean [SD] age 65.4 [10.2], 57% females; Table 1). Six participants were classified as CU, 97 as MCI, and 47 as dementia due to AD. In the MCSA sample, 18 participants had MCI and 26 had dementia with atypical AD clinical features. A flow chart detailing reasons for participant exclusion and the derivation of the final analytical samples is provided in Supplementary Figure S2.

**Table 1:** Demographic, clinical, and biomarker characteristics of the participants.

	ADNI (n = 150)	MCSA (n = 44)
Age (years), mean (SD)	75.0 (7.7)	65.4 (10.2)
Female, n (%)	65 (43%)	25 (57%)
Male, n (%)	85 (57%)	19 (43%)
Years of education, mean (SD)	16.1 (2.5)	16.0 (3.7)
Clinical diagnosis, n (%)^a^	6 Aβ+ CU (4%) 97 Aβ+ MCI (65%) 47 Aβ+ dementia (31%)	18 Aβ+ MCI (41%) 26 Aβ+ dementia (59%)
MMSE, mean (SD)	26.0 (3.4)	21.2 (6.1)
APOE ε4 carriers, n (%)	89 (59%)	31 (70%)

AD: Alzheimer's disease; APOE: apolipoprotein E; CU: cognitively unimpaired; MCI: mild cognitive impairment; MMSE: Mini-Mental State Examination; SD: standard deviation.

a For the MCSA subset, the displayed diagnoses represent the primary clinical hypothesis among the differential diagnoses formulated prior to biomarker and [^18^F]FDG-PET assessments.

The mean (SD) time between tau-PET and [^18^F]FDG-PET assessments was 0.8 (1.2) and 8.8 (7.9) months for the ADNI and MCSA samples, respectively.

### Tau extent predicts quantitative AD-specific hypometabolism better than tau load

**Fig. 1:**
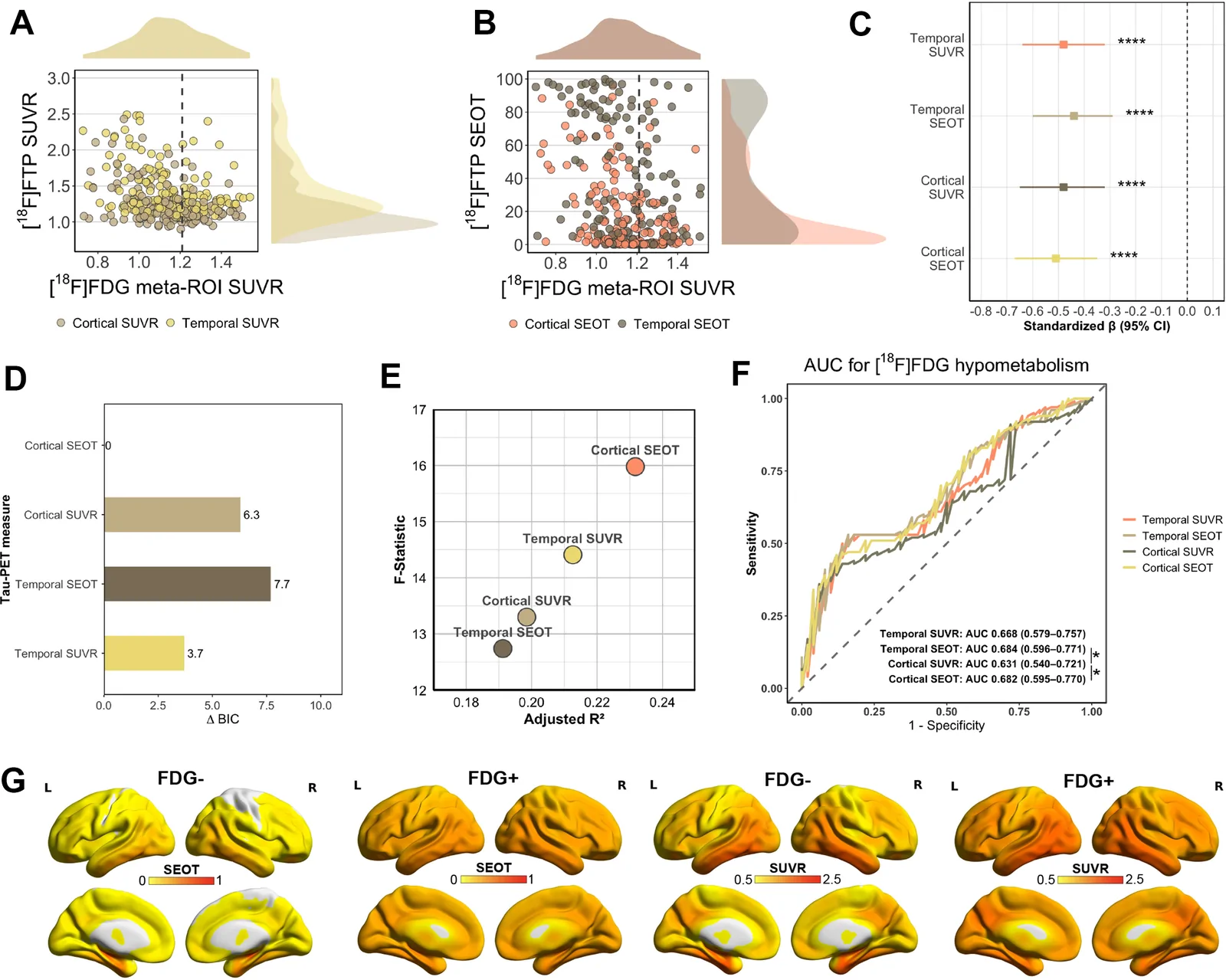
**Tau extent outperforms tau load for predicting AD-associated hypometabolism.** In **A** FDG-PET meta-ROI SUVR is plotted against tau load (tau-PET SUVR), and in **B** against tau extent (SEOT). **C** shows standardised effect sizes with 95% CI from separate age- and sex-adjusted models predicting [^18^F]FDG-PET meta-ROI SUVR from each tau metric. Model comparison in **D** (ΔBIC relative to the best model) favours cortical SEOT models, while overall fit indices in **E** (F-statistic and adjusted R^2^) reinforce this advantage. In **F** ROC curves compare the ability of the tau metrics to classify quantitatively-defined [^18^F]FDG-PET hypometabolism, with AUCs and corresponding 95% CIs shown. **G** presents group-average cortical maps of tau-PET SUVR and SEOT for participants with (FDG+) and without (FDG−) AD-related hypometabolism. Voxelwise SEOT frequency maps were generated by averaging participant-specific binary tau abnormality maps, such that voxel intensities represent the proportion of participants with suprathreshold tau-PET signal at each location. Maps were visualised using BrainNet Viewer. All panels include 150 ADNI participants; in **F** and **G**, 100 participants were FDG+ and 50 were FDG−. ∗p < 0.05, ∗∗p < 0.01, ∗∗∗p < 0.001,∗∗∗∗p < 0.0001.

We compared different tau-PET summary measures (cortical and temporal SUVR and SEOT) regarding their association with the [^18^F]FDG-PET meta-ROI SUVR; all were significantly associated (p < 0.0001) with hypometabolism (Fig. 1A–C). The model with cortical SEOT as the main predictor showed the best BIC (ΔBIC >2), F-statistic, and adjusted R^2^ values (Fig. 1D and E). We also tested models adjusting for Aβ-PET load and CSF Aβ_42_/_40_ ratio, whose inclusion resulted in worse model fit and no significant associations for the Aβ predictors (all p > 0.05; Supplementary Table S3). In sensitivity analyses evaluating SEOT derived using z-scoring thresholds, models adjusted for age and sex also showed the best fit for cortical SEOT derivations (Supplementary Table S4). Similar findings were observed in models further adjusted for education, APOEε4 status and clinical diagnosis (Supplementary Table S5).

In ADNI, 100 (67%) participants were classified as having [^18^F]FDG-PET hypometabolic pattern (FDG+). AUC values of SEOT were numerically higher for predicting FDG + status, and both SEOT metrics showed significantly better AUC than whole-cortex SUVR (all p < 0.05; Fig. 1F). No other significant differences were observed. Consistent findings were observed in sensitivity analyses using z-scored SEOT derivations (Supplementary Table S6). Fig. 1G displays average SEOT and SUVR maps for FDG- and FDG + groups.

### Tau extent is more closely associated with local hypometabolism than tau load

**Fig. 2:**
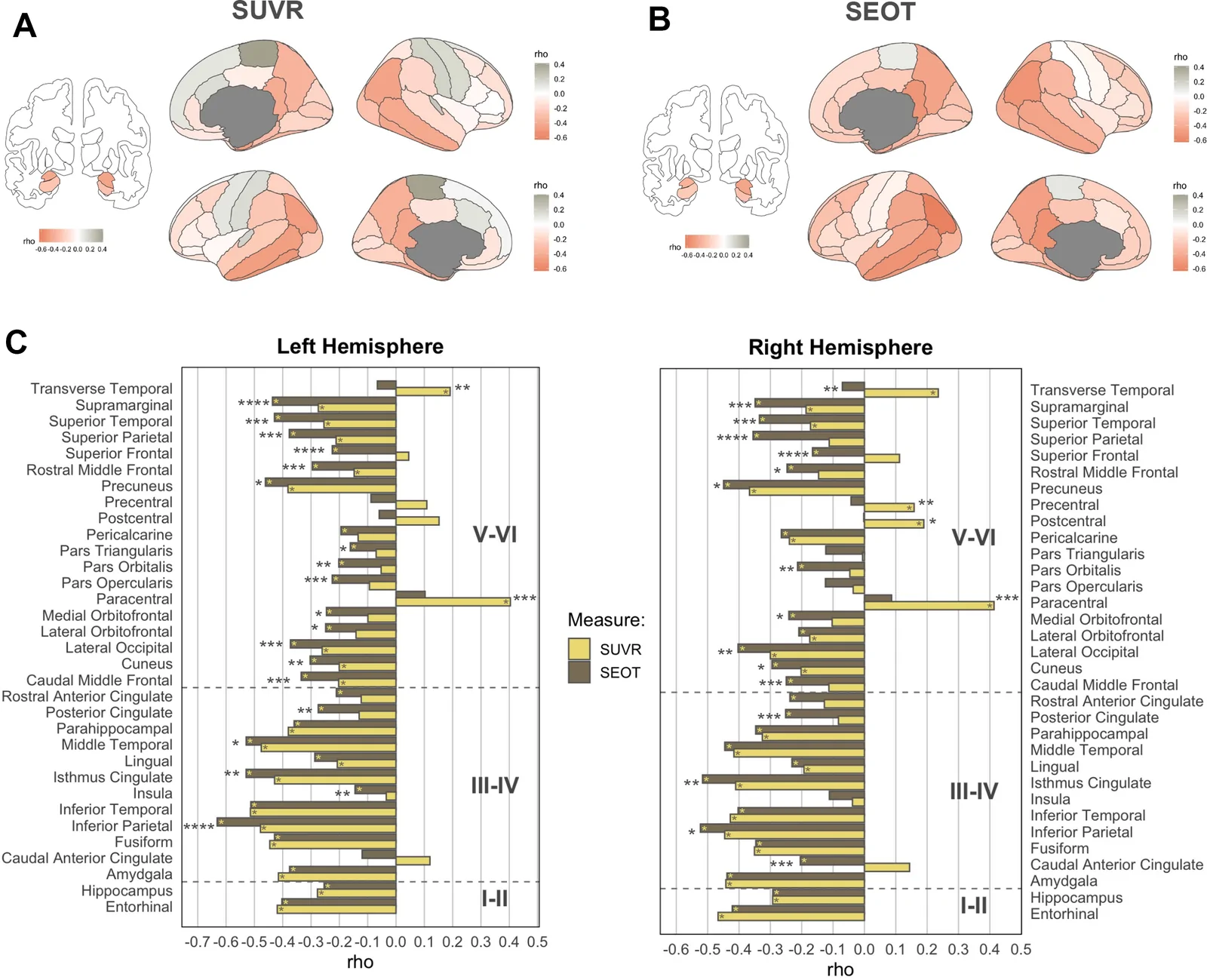
**Tau extent correlates better with regional brain metabolism than tau load. A** and **B** show brain surface maps of regional Spearman's correlations between tau-PET SUVR (**A**) or SEOT (**B**) and local [^18^F]FDG-PET SUVR, displayed using the *ggseg* R package. The same correlation coefficients are summarised in the bar plot in **C**. Statistical significance for individual rho coefficients (∗ for p < 0.05) is shown inside each bar, while p-values for the comparison between tau-PET SUVR and SEOT coefficients are displayed above the corresponding bar pairs when significant. All panels include 150 ADNI participants. ∗p < 0.05, ∗∗p < 0.01, ∗∗∗p < 0.001, ∗∗∗∗p < 0.0001.

Across DKT regions, SEOT showed similar or stronger associations with local hypometabolism than tau load (Fig. 2A–C). The strongest advantage for SEOT was observed in widespread bilateral association cortices and paralimbic areas, as well as in select left-hemisphere temporal and frontal regions. In a small number of areas, including the bilateral rostral anterior cingulate and left pericalcarine, only SEOT was associated with hypometabolism.

In contrast, both metrics correlated similarly with [^18^F]FDG-PET signal in several early-stage temporolimbic regions, such as the entorhinal cortex and hippocampus. Some sensorimotor areas (e.g., right precentral and postcentral gyri) showed significant positive correlations for tau load only.

### Distributed cortical tau patterns predict AD-related hypometabolic signatures

**Fig. 3:**
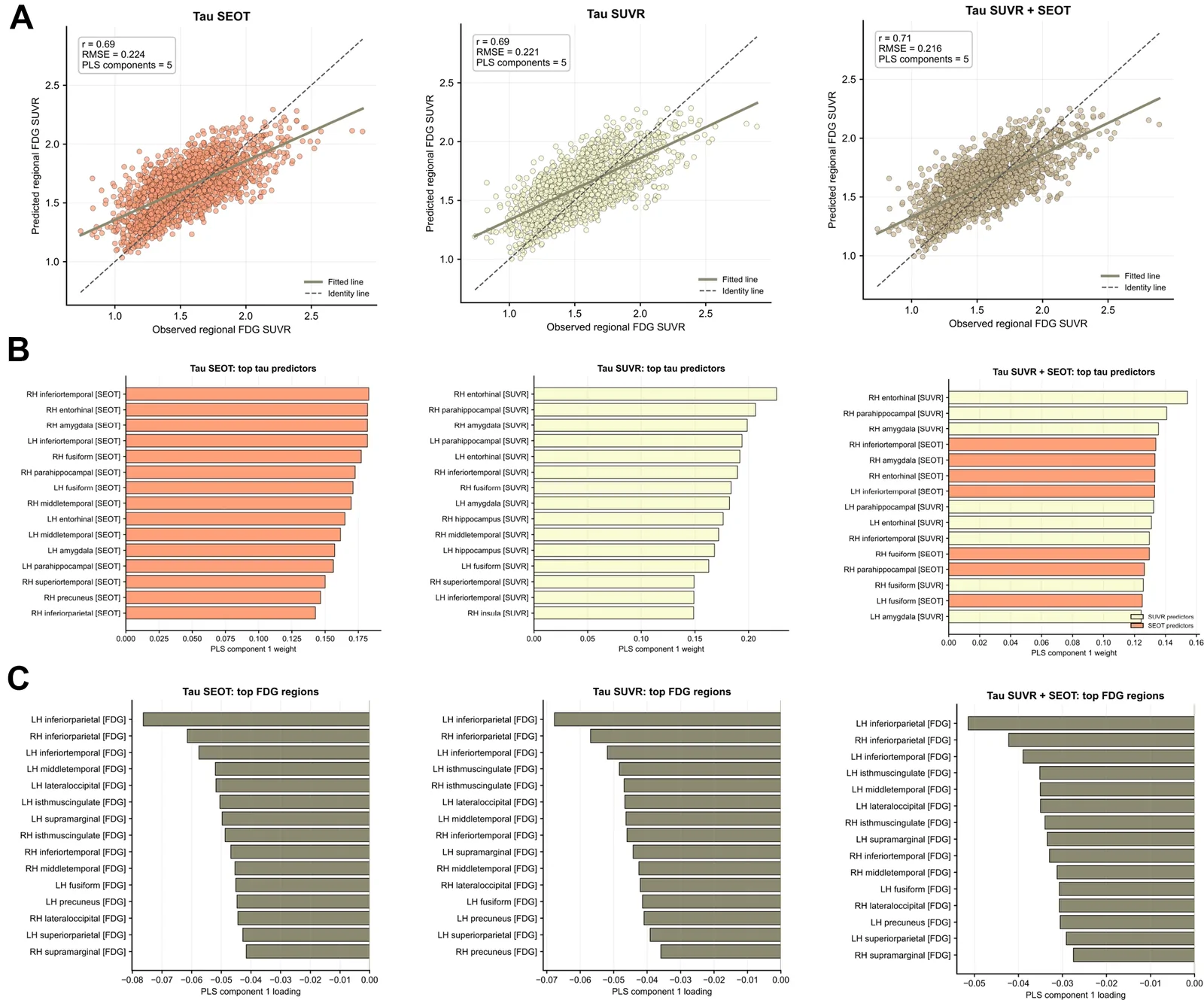
**Partial least squares (PLS) prediction of brain metabolic patterns from distributed cortical tau-PET measures. A** shows scatter plots comparing observed and PLS-predicted regional FDG SUVR values for models using tau SEOT, tau SUVR, and combined tau SUVR + SEOT predictors. Each point represents a regional FDG measurement across participants. Solid lines indicate fitted regression lines, whereas dashed lines represent the identity line. Model performance metrics, including Pearson correlation coefficient (r), root mean squared error (RMSE), and the optimal number of PLS components, are displayed within each panel. **B** and **C** show, respectively, the regional tau predictors and FDG regional loadings with the strongest contributions to the first latent PLS component across the tau SUVR, SEOT, and combined SUVR + SEOT models. All panels include 150 ADNI participants.

PLS regression demonstrated that distributed cortical tau patterns strongly predicted [^18^F]FDG-PET signatures (Fig. 3A). Separate tau load and SEOT models achieved comparable predictive performance (r = 0.69 for both models), whereas the combined tau load + SEOT model yielded the strongest prediction performance (r = 0.71) and lowest prediction error (RMSE = 0.216). Cross-validation analyses demonstrated that predictive performance peaked at five latent components across all models (Supplementary Figure S3).

Inspection of the predictor-side PLS components revealed that the strongest tau contributors were consistently localised to temporolimbic regions, with additional contributions from temporoparietal regions (Fig. 3B). These regions mostly spanned early-to-intermediate Braak territories and showed substantial overlap across tau load and SEOT models. Nonetheless, select later-stage Braak regions, such as the praecuneus, showed greater relative importance in the SEOT model, whereas hippocampal contributions were more prominent in tau load models. In the combined model, both tau load-derived and SEOT-derived predictors contributed prominently to the latent component.

On the outcome side, [^18^F]FDG-PET loadings revealed consistent AD-related metabolic signatures across models, predominantly involving temporoparietal and posterior associative cortices (Fig. 3C), showing limited spatial overlap with the principal tau predictors.

### Tau extent tracks network-level hypometabolism better than tau load

**Fig. 4:**
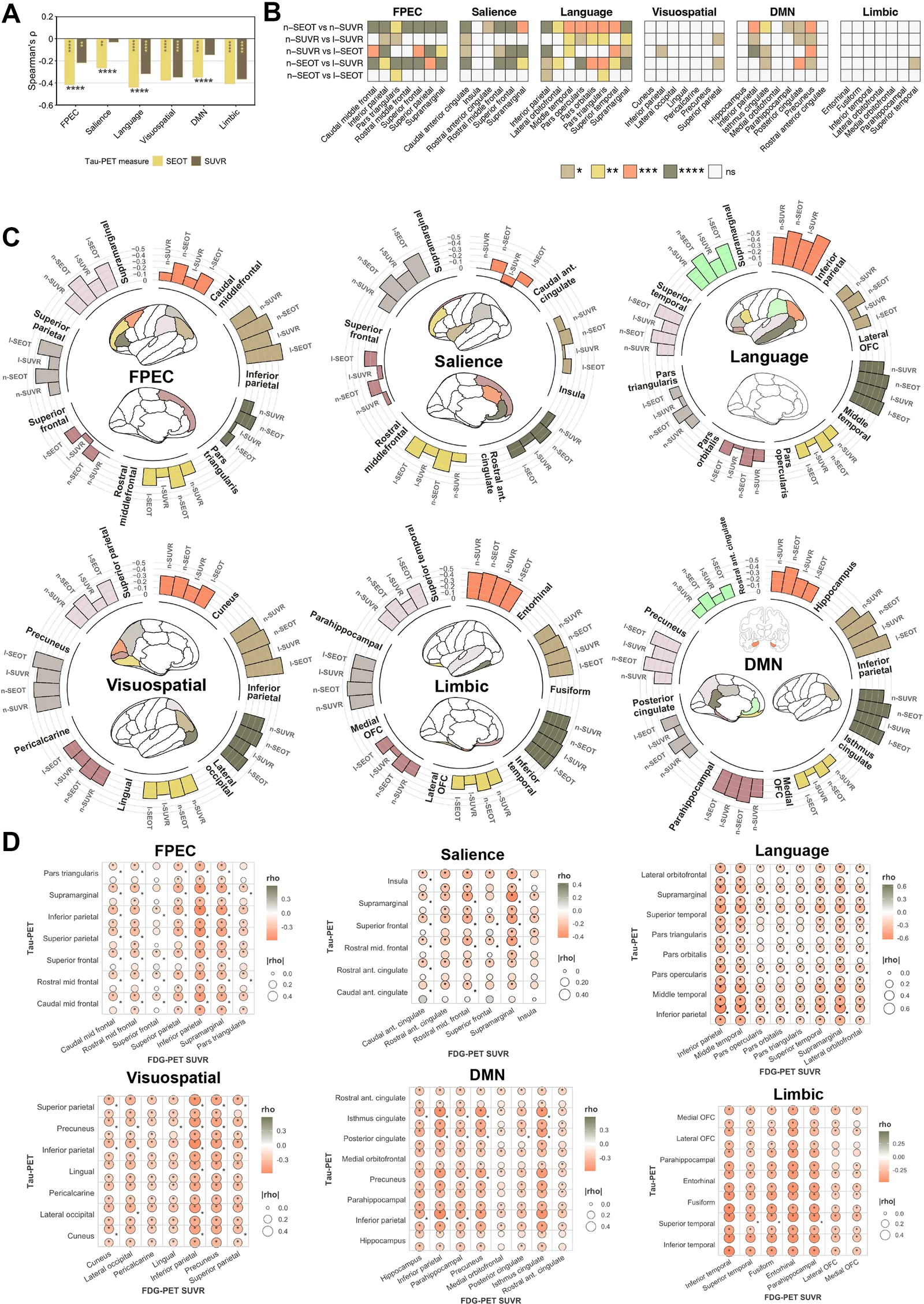
**Tau extent outperforms tau load in tracking brain hypometabolism in AD-related brain networks. A** shows the rho coefficients for the correlations between whole-network tau load or extent and whole-network FDG-PET signal, with statistical significance for individual rho values displayed inside the bars and p-values for tau-PET SUVR vs SEOT comparisons shown above bar pairs when significant. **B** and **C** compare the correlations of local tau-PET SUVR (l-SUVR) and SEOT (l-SEOT) versus whole-network tau-PET SUVR (n-SUVR) and SEOT (n-SEOT) with local [^18^F]FDG-PET signal for each core or supporting region within each network. **B** shows heatmaps summarising the statistical comparison of the correlation coefficients, whose strength is illustrated in the radar plots in **C**. **D** presents the rho coefficients of the correlation between SEOT or tau load and [^18^F]FDG-PET SUVR across network nodes. Each cell contains a top (SEOT) and a bottom (tau load) circle representing the correlations between the tau-PET metrics and [^18^F]FDG-PET SUVR in regions composing each network (i.e., nodes). Circle size reflects the absolute magnitude of the rho coefficient, while the colour scale indicates its signed value and direction, as shown on the right of each graph. Statistical significance (∗ for any p < 0.05) for individual rho values is displayed inside the circles, and p-values for tau load vs SEOT comparisons are shown to the right of each circle pair when significant. All panels include 150 ADNI participants. ∗p < 0.05, ∗∗p < 0.01, ∗∗∗p < 0.001, ∗∗∗∗p < 0.0001.

Across six large-scale functional networks, SEOT values extracted from whole-network masks correlated more strongly with [^18^F]FDG-PET SUVR measured within the same network than did tau-PET SUVR from those territories in four networks: FPEC, Language, Salience, and DMN (p < 0.0001; Fig. 4A). In the Limbic and Visuospatial network, the difference between metrics was not statistically significant, although the SEOT coefficient was numerically larger in magnitude.

Sensitivity analyses using SEOT z-scoring thresholds (2, 2.5, and 3 SD) showed patterns consistent with the primary analyses (Supplementary Table S7). Across most thresholds, SEOT demonstrated stronger associations with within-network [^18^F]FDG-PET SUVR than regional tau load in the DMN, FPEC, Language, and Salience networks, whereas differences remained non-significant in the Visuospatial and Limbic networks despite numerically larger SEOT coefficients. These findings were largely preserved after additional adjustment for education, APOEε4 status, and clinical diagnosis (Supplementary Table S8).

When relating network-level tau metrics to regional hypometabolism, network SEOT showed stronger correlations than network tau load across all FPEC and Language regions and across most DMN and Salience regions (Fig. 4B–C). No significant differences emerged in the Visuospatial or Limbic networks, although SEOT coefficients were numerically larger in all cases. Network SEOT outperformed local tau load most consistently in the FPEC and Language networks, while also showing considerable advantages in the DMN and Salience networks. Comparisons between network- and local-level SEOT associations revealed only three significant regional differences. By contrast, local SEOT outperformed network-level tau load associations in 17/43 comparisons. Finally, significant differences between network- and local-level tau load associations with regional hypometabolism were observed in 12/43 comparisons, most favouring network load. Importantly, tau load metrics did not outperform SEOT metrics in any comparison.

Node-to-node analyses showed that correlations between SEOT and [^18^F]FDG-PET SUVR in distant but functionally interconnected regions were either similar to or higher than those observed for tau load and [^18^F]FDG-PET SUVR (Fig. 4D). A stronger performance of SEOT was observed in the FPEC and Language networks, where SEOT outperformed tau load across most node-to-node interconnections.

### Tau extent and load are complementary predictors of voxel-wise global hypometabolism

**Fig. 5:**
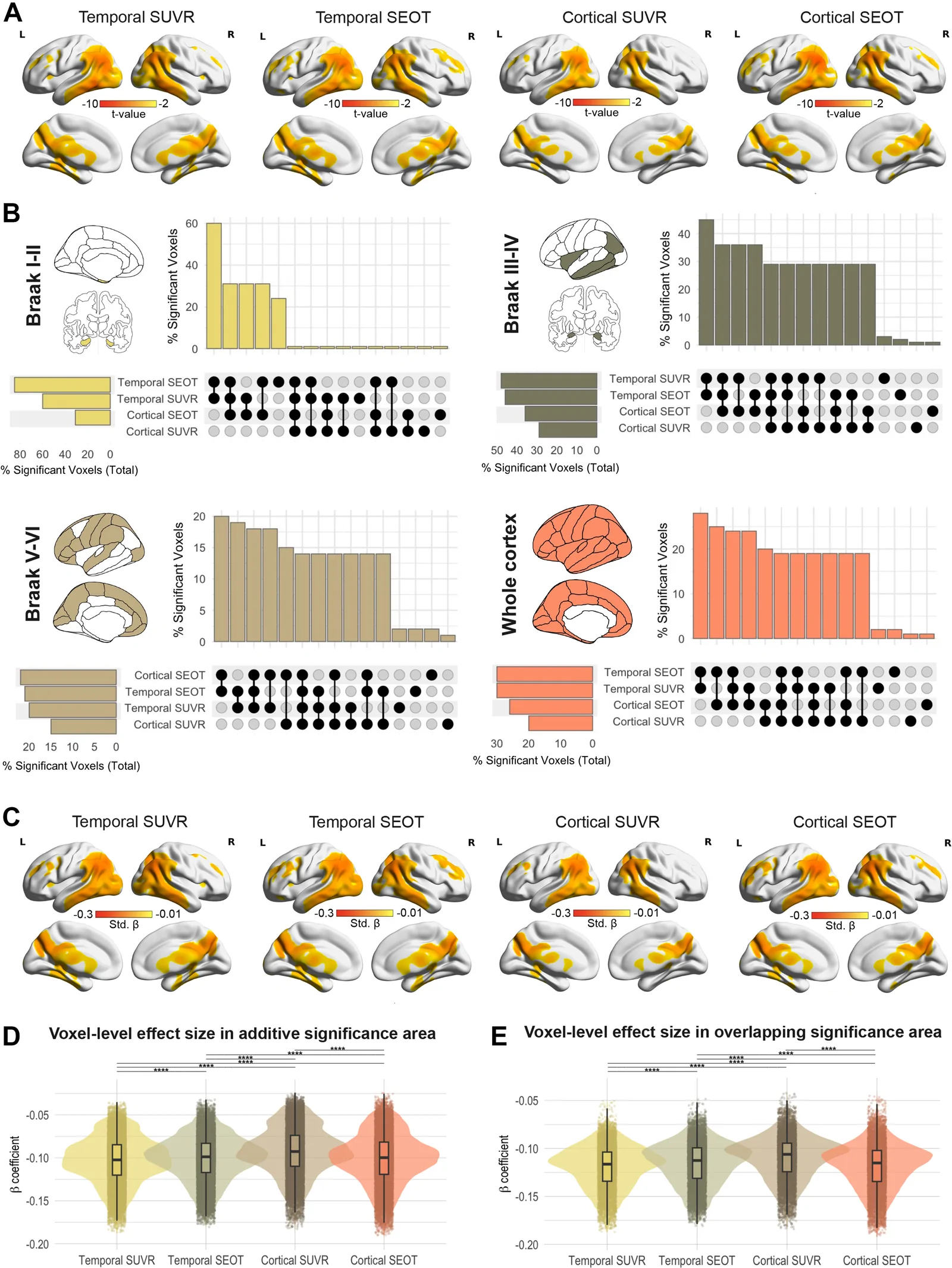
**Tau extent and load provide complementary explanations of global brain hypometabolism. A** shows t-maps from voxelwise linear regressions between tau-PET metrics (predictors) and FDG-PET (outcome), adjusted for age and sex. **B** illustrates the overlap between significant voxels from these t-maps and anatomical masks corresponding to Braak I–II, Braak III–IV, Braak V–VI, and the whole cortex. In each mask, horizontal bars indicate the total percentage of significant voxels (from each tau-PET t-map) overlapping with that mask. Vertical bars indicate the proportion of significant voxels within ROI masks that were either uniquely present in a single t-map (lone dots) or shared across multiple t-maps (connected dots, representing the percentage of voxels significant for two or more predictors at the same time). **C** displays standardised beta coefficient maps from the same voxelwise regressions, with values shown only where associations were statistically significant in the t-maps. **D** and **E** present combined violin and box plots with individual data points displayed, showing standardised β values extracted for each predictor in additive significance areas (where at least one predictor was significant; **D**) and in overlapping significance areas (where all predictors were significant; **E**). Boxes show the interquartile range and median, whiskers extend to 1.5 times the interquartile range, and points represent individual voxels. All panels include 150 ADNI participants.

Voxelwise linear regression analyses using temporal and cortical SUVR and SEOT as main predictors revealed that significant voxels were predominantly located in bilateral association cortices, encompassing temporal, parietal, and frontal regions, with additional clusters in medial occipital and paralimbic areas (Fig. 5A).

Temporal SEOT showed the greatest proportion of significant voxels within early Braak regions (85%), with over 20% significant only for this predictor (Fig. 5B). Cortical SUVR showed no significant associations in Braak I–II. Temporal SUVR and SEOT achieved the greatest overlap with Braak III–IV (48% and 46%, respectively), with 45% shared significant voxels. Cortical SEOT showed the largest extent of significant voxels in Braak V–VI (22%). Temporal SUVR and SEOT showed the greatest whole-cortex overlap (30%), whereas cortical SUVR had the lowest (20%).

Analysis of voxel-level standardised β coefficients from additive and overlapping significance maps (Fig. 5C–E) showed that the largest effect magnitudes, reflecting more negative associations, followed the rank order: temporal SUVR > cortical SEOT > temporal SEOT > cortical SUVR (all p < 0.0001).

In voxelwise models simultaneously including SEOT and SUVR predictors, most associations were sparse, suggesting substantial shared variance (Supplementary Figure S4). However, cortical SEOT retained associations with hypometabolism after accounting for cortical SUVR, particularly within bilateral association cortices involving inferior parietal/angular, posterior lateral temporal, and dorsolateral prefrontal regions. Associations for temporal SUVR after adjustment for SEOT measures were limited to small temporal clusters, whereas cortical SUVR yielded no significant clusters. Cortical SEOT retained focal temporoparietal junction clusters after adjustment for temporal SUVR, while temporal SEOT retained a small parietal cluster after adjustment for cortical SUVR.

### Stage-dependent associations between tau-PET and brain glucose hypometabolism

In pre-dementia participants, all tau-PET metrics were significantly associated with lower [^18^F]FDG-PET meta-ROI SUVR, with temporal SEOT yielding the greatest effect sizes and model fit across SEOT derivations (Supplementary Table S9). In the dementia subgroup, no tau-PET predictors reached statistical significance (Supplementary Table S10).

In voxelwise analysis, all tau-PET metrics were associated with [^18^F]FDG-PET hypometabolism predominantly within bilateral temporoparietal association cortices in pre-dementia participants (Supplementary Figure S5A), with slightly more extensive clusters for temporal metrics. In dementia participants, associations were markedly attenuated, with focal significant clusters only for temporal SUVR and cortical SEOT (Supplementary Figure S5B) involving the right posterior inferior temporal cortex.

### Tau extent outperforms tau load in predicting visually-assigned AD-specific hypometabolism in atypical AD

**Fig. 6:**
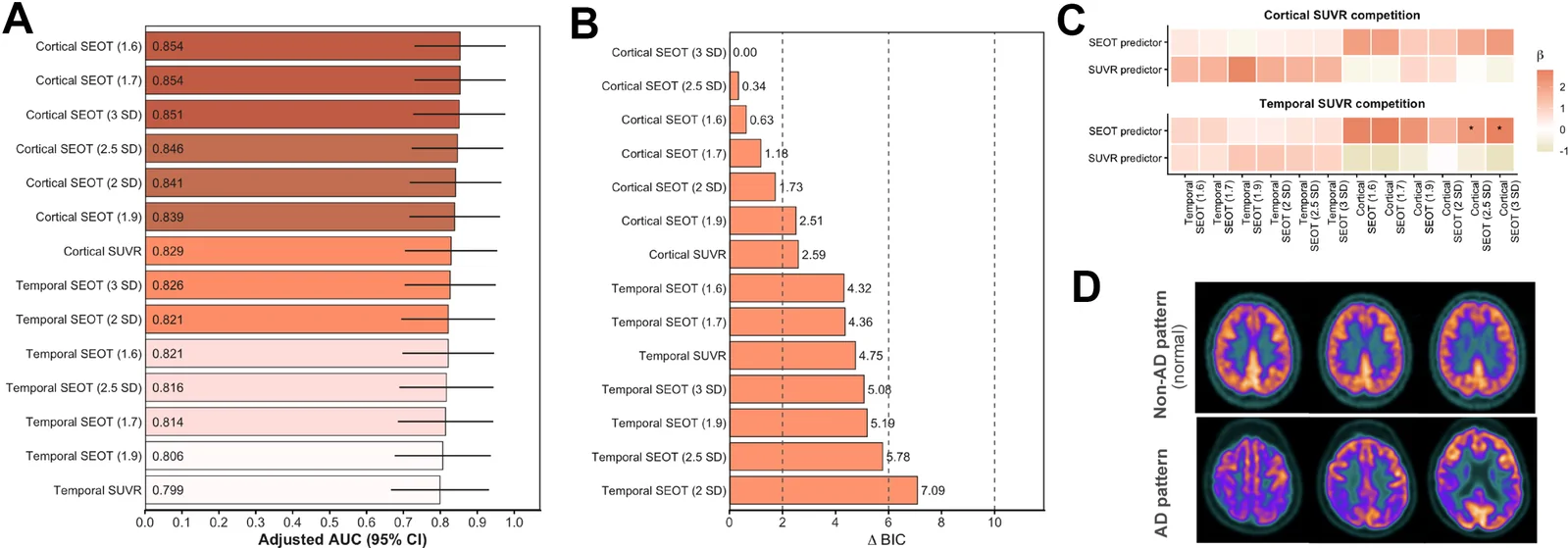
**Cortical tau extent improves the identification of visually assessed [^18^F]FDG-PET AD-like patterns of hypometabolism. A** presents bar plots of AUC values with 95% CIs for the ability of each tau-PET metric to discriminate between AD-like and non-AD hypometabolic patterns. All AUC estimates were derived from logistic regression models adjusted for the interval between [^18^F]FDG-PET and tau-PET scans. Pairwise comparisons of AUC values using DeLong's test revealed no significant differences between metrics. **B** displays ΔBIC values relative to the best-performing model (ΔBIC = 0). **C** shows a heatmap of standardised beta coefficients from competitive logistic regression models simultaneously including SEOT and SUVR predictors for the prediction of [^18^F]FDG-PET classification status. Symbols within cells indicate predictors that remained significant after adjustment for the competing tau-PET metric. **D** presents representative [^18^F]FDG-PET scans from participants in each classification group. Analyses include 44 MCSA participants: 31 with an AD-like hypometabolic pattern and 13 with a non-AD pattern. ∗p < 0.05.

Among MCSA participants, 31 (70%) were classified as having an AD hypometabolic pattern and 13 (30%) as non-AD. Whole-cortex tau-PET measures demonstrated numerically higher AUC values than temporal metrics, with cortical SEOT yielding the highest values across all derivations (Fig. 6A). DeLong comparisons revealed no statistically significant AUC differences. Cortical SEOT models demonstrated the best BIC, followed by cortical SUVR, temporal SUVR, and temporal SEOT derivations (Fig. 6B). Cortical SEOT derived using voxelwise 2.5 and 3 SD thresholds demonstrated considerably better model fit than cortical SUVR (ΔBIC > 2). In competitive logistic regressions simultaneously including SEOT and SUVR predictors, most associations were attenuated (Fig. 6C). However, cortical SEOT derived using voxelwise 2.5 and 3 SD thresholds remained independently associated with AD-like hypometabolism even when competing against temporal SUVR (all p < 0.05). Representative cases are shown in Fig. 6D.

### Tau extent retains independent associations with cognition after accounting for FDG-PET and tau load

**Fig. 7:**
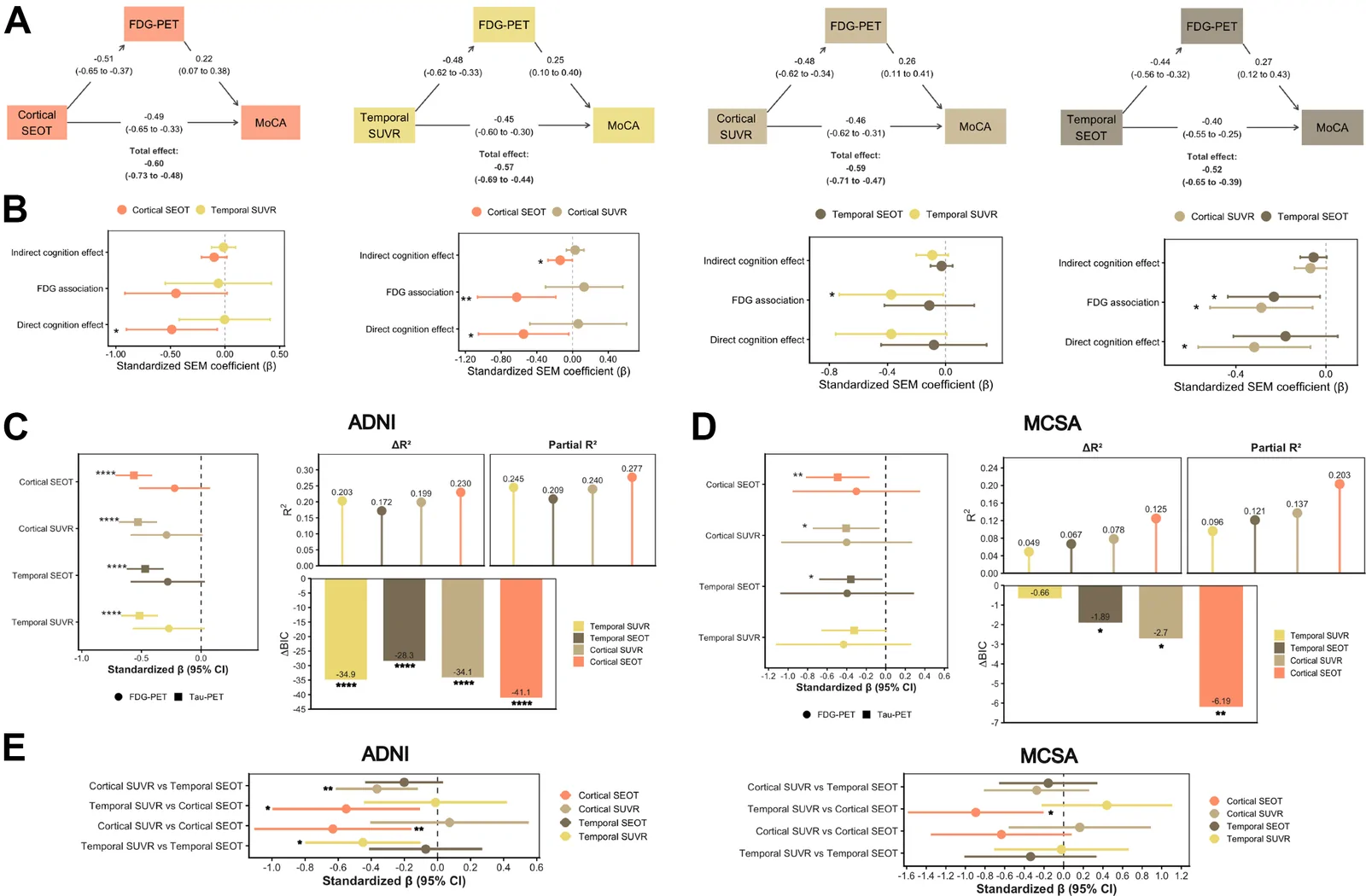
**Tau extent retains independent associations with cognition after accounting for [^18^F]FDG-PET hypometabolism and tau load. A** presents SEMs examining associations between individual tau-PET metrics, [^18^F]FDG-PET meta-ROI SUVR, and MoCA scores in ADNI. Standardised path coefficients (β) and 95% CI are displayed for each pathway, together with the total effect of each tau-PET metric on cognition. **B** shows SEM coefficients from competitive models jointly including SEOT and SUVR metrics to assess their independent contributions to cognition and [^18^F]FDG-PET after accounting for shared variance between tau measures. Direct and indirect cognition effects correspond respectively to direct tau–MoCA associations and [^18^F]FDG-mediated pathways. “FDG association” represents the relationship between tau-PET metrics and [^18^F]FDG-PET meta-ROI SUVR. Error bars represent 95% CI. **C** and **D** show hierarchical regression analyses evaluating the additional explanatory value of tau-PET metrics for MoCA scores beyond binary [^18^F]FDG-PET status in the ADNI (C) and MCSA (D) datasets. Left plots display β coefficients and 95% CI for [^18^F]FDG-PET abnormality and tau-PET predictors. Upper right plots show changes in explained variance (ΔR^2^) and partial R^2^ values after adding each tau-PET metric to the base model containing demographics and [^18^F]FDG-PET status. Lower right plots display ΔBIC values relative to the [^18^F]FDG-PET-only model, with ANOVA p-values versus the base model shown at the bottom of each bar. **E** presents β coefficients and 95% CI from competitive linear regression models jointly including SEOT and SUVR predictors to assess their independent associations with MoCA beyond [^18^F]FDG-PET status. All models were adjusted for age, sex, and years of education. MCSA models were additionally adjusted for the interval between [^18^F]FDG-PET and tau-PET acquisition. The ADNI and MCSA analyses include 150 and 44 participants, respectively. ∗p < 0.05; ∗∗p < 0.01; ∗∗∗∗p < 0.0001.

SEM analyses demonstrated that all tau-PET metrics were associated with lower MoCA scores both directly and indirectly through [^18^F]FDG-PET hypometabolism (Fig. 7A). Across individual models, cortical SEOT showed the numerically strongest association with MoCA, followed by cortical SUVR, temporal SUVR, and temporal SEOT. Direct associations with cognition were stronger than FDG-mediated effects across all models, while FDG-PET mediation effects remained modest but significant.

Competitive SEMs revealed differential patterns between tau extent and load metrics. In the cortical SEOT versus temporal SUVR model, only cortical SEOT retained significant direct associations with cognition (Fig. 7B). In the cortical SEOT versus cortical SUVR model, only cortical SEOT remained significantly associated with [^18^F]FDG-PET and cognition, both directly and indirectly through [^18^F]FDG-PET. When temporal SEOT and temporal SUVR were jointly modelled, temporal SUVR retained significant direct [^18^F]FDG-related associations, whereas temporal SEOT showed no independent associations. In the cortical SUVR versus temporal SEOT model, cortical SUVR remained significantly associated with [^18^F]FDG-PET and with cognition through the direct pathway, while temporal SEOT retained associations with [^18^F]FDG-PET only.

Hierarchical regression analyses demonstrated that tau-PET metrics explained additional cognitive variance beyond [^18^F]FDG-PET status (Fig. 7C and D). Across cohorts, cortical SEOT showed the strongest performance, yielding the largest ΔR^2^, highest partial R^2^, and greatest improvement in model fit relative to the FDG-only model. All other tau-PET metrics also improved models in ADNI. In MCSA, temporal SUVR did not reach statistical significance.

Models jointly including SEOT and tau load predictors showed that cortical SEOT retained significant associations with lower MoCA after adjusting for cortical SUVR in both datasets and after adjusting for temporal SUVR in MCSA participants (Fig. 7E). Both SUVR metrics retained independent associations with MoCA after adjustment for temporal SEOT in ADNI.

## Discussion

In this study, we investigated the relationship between tau pathology and brain glucose metabolism in two distinct cohorts. We compared two tau-PET metrics, tau load (regional SUVR) and tau extent (SEOT), to determine how each reflects downstream neurodegenerative processes in AD. Across spatial scales (i.e., from signature meta-ROIs to regional, network, and voxelwise analyses), SEOT provided complementary, independent, and in several cases stronger predictive value than tau load for brain hypometabolism. These findings extend previous work demonstrating that tau-PET extent provides information beyond conventional tau load for explaining neural and clinical degeneration in AD^8^, ^9^, ^10^^,^^31^^,^^33^ and align with histopathological evidence showing that combining measures of tau density and spatial extent improves disease severity characterisation.^49^

Our findings indicated cortical SEOT as the strongest predictor of cognition, corroborating prior reports showing stronger cognitive associations for tau extent than tau load.^8^, ^9^, ^10^^,^^31^ Importantly, global cognitive impairment becomes more prominent once tau pathology reaches PET-Braak IV–VI stages.^50^ Consistent with this, cortical SEOT showed the broadest hypometabolic associations in late Braak regions, suggesting that this metric better captures neocortical dysfunction extending beyond the Braak III–IV territories covered by the [^18^F]FDG-PET meta-ROI^30^^,^^34^ and potentially contributing to cognitive dysfunction. At the same time, Boccalini et al. demonstrated that [^18^F]FDG-PET hypometabolism only partially mediates the association between tau-PET and cognition, with some cortical regions showing direct tau-related cognitive effects independent of measurable hypometabolism.^51^ By encompassing the whole cortex, cortical SEOT may capture clinically relevant tau involvement extending beyond regions with overt hypometabolism. Moreover, because SEOT emerges at relatively low levels of tau burden,^10^ it may be particularly sensitive to the early neocortical dissemination of tau pathology. Together, these findings suggest that global cortical assessment of tau extent may complement conventional tau-PET approaches by capturing both regions where tau overlaps with [^18^F]FDG-PET-defined neurodegeneration and spatially diffuse tau-positive territories whose cognitive effects are not fully explained by measurable hypometabolism.

Tau-related cognitive effects not mediated by [^18^F]FDG-PET may reflect additional downstream mechanisms, including neuroinflammation.^52^ Because [^18^F]FDG-PET may capture not only neuronal dysfunction but also altered glial metabolic activity,^53^^,^^54^ part of the observed FDG-mediated effects may likewise reflect inflammatory processes associated with tau pathology. In this context, SEOT may also capture aspects of neuroinflammation previously associated with tau load.^55^^,^^56^ This dual signal may help explain the positive tau-FDG associations observed in a small number of late Braak regions. Indeed, previous work has shown that higher glial fibrillary acidic protein, a marker of astrocytic reactivity, is associated with increased glucose consumption early in the AD continuum, particularly before substantial tau accumulation is present.^54^ Thus, the positive tau–FDG associations may reflect early glial-metabolic responses accompanying initial tau spread into regions with relatively low tau burden, in which astrocytic activation may predominate before the emergence of the hypometabolic patterns typically associated with established tau pathology. Future studies should investigate the relative contributions of neuroinflammatory and neurodegenerative processes to [^18^F]FDG-PET signal across stages of tau accumulation.

Our findings have important clinical implications. In a cohort enriched for atypical phenotypes, in which non-canonical tau- and [^18^F]FDG-PET patterns are expected,^37^^,^^40^ cortical SEOT showed the best performance for identifying AD-like hypometabolism and predicting cognitive impairment. Unlike traditional staging strategies based on fixed ROIs^5^^,^^32^ or PET-Braak staging,^4^^,^^50^ SEOT may reliably quantify disease burden without presupposing a canonical topography, making it particularly valuable for detecting non-conformant tau patterns.^57^ Moreover, whole-cortex SEOT provided complementary information to temporal load for predicting hypometabolism, especially in Braak V–VI regions often affected earlier in atypical phenotypes such as PCA.^58^ These findings align with reports showing that whole-cortex SEOT accurately identifies neuropathological Braak V–VI tau NFT, performing comparably or better than tau-PET visual reads and SUVR metrics.^31^ Together, the spatial agnosticism and strong neocortical sensitivity of whole-cortex SEOT likely underlie its good performance in atypical AD.

Although cortical SEOT better captured the extent of neocortical metabolic dysfunction, voxelwise analyses showed that temporal tau load yielded the strongest effect magnitudes. Machine learning analyses further demonstrated that combined tau extent and load models provided the strongest prediction of metabolic signatures, supporting partially non-redundant contributions. Together, these findings suggest that tau load and extent capture complementary dimensions of tau-mediated neurodegeneration, characterised by abnormal tau detaching from microtubules, mislocalising to pre- and postsynaptic compartments, impairing axonal transport and mitochondrial function, disrupting neuronal signalling, and ultimately promoting synaptic and neuronal injury.^59^ At the systems level, tau accumulation has been associated with the brain's functional network architecture, consistent with the hypothesis that the spatial progression of tau pathology occurs preferentially across interconnected brain regions.^60^^,^^61^ In our study, SEOT tracked hypometabolism more accurately across large-scale functional networks than tau load, which may dilute sparse but biologically meaningful focal pathology by averaging signal intensity across broad cortical territories.^6^ SEOT also better predicted hypometabolism in distant but connected nodes, extending previous research using load-based metrics.^7^^,^^62^ Collectively, these findings suggest that SEOT offers a more precise proxy of tau distribution across networks, providing complementary information to conventional tau load measures.

A possible explanation for the strong network-level performance of SEOT is its greater sensitivity to early regional tau involvement.^10^ Tau load, in turn, may preferentially reflect tau burden at later phases of regional occupation by tau pathology.^10^ Because our samples mostly included cognitively impaired individuals, who frequently exhibit intermediate or advanced tau-PET stages,^4^^,^^50^ earlier Braak regions may already have shown substantial tau involvement, a stage at which tau load and extent may perform similarly in tracking regional tau burden.^10^ This may explain their comparable performance in early Braak regions and in networks representing the prevalent initial hubs of tau propagation, such as the limbic network.^63^ Meanwhile, neocortical tau propagation was likely still active in many individuals,^64^ a phase in which SEOT may be particularly sensitive because it demonstrates greater variance at lower regional tau-PET burden.^10^ This may help explain SEOT's superiority in regions affected later in disease progression, which likely exhibited lower tau burden than regions involved earlier in AD. Together, these findings support the possibility that tau load and SEOT may optimally capture different phases of tau progression within the same brain region.

This temporal ordering between SEOT, tau load, and brain hypometabolism was further supported by subgroup analyses showing superior performance of temporal SEOT for predicting hypometabolism in pre-dementia individuals. This aligns with findings demonstrating stronger associations between SEOT and cognitive changes in preclinical and prodromal populations, particularly in medial temporal regions.^10^ In the dementia subgroup, no tau-PET measure significantly predicted [^18^F]FDG-PET meta-ROI signal,^30^^,^^34^ although voxelwise analyses demonstrated that temporal SUVR and cortical SEOT still tracked hypometabolism in the inferior temporal gyrus. This suggests a more regionally restricted relationship between interindividual tau burden and metabolic dysfunction during late disease, highlighting the potential need for [^18^F]FDG-PET outcomes better suited to detect residual metabolic heterogeneity in dementia phases. This lower variability may reflect a previously described plateau effect of [^18^F]FDG-PET measures,^65^ as well as the dissociation between tau pathology and neurodegeneration observed in AD,^66^ which may become more pronounced as disease severity progresses.

Several limitations must be acknowledged. First, tau-PET tracer harmonisation frameworks^67^ were not applied. Although cohorts were analysed independently using tracer-specific thresholds, the absence of harmonisation may limit cross-tracer generalisability, particularly for voxelwise spatial extent metrics not yet validated within harmonisation frameworks. Second, network-level analyses relied on previously published functional network templates,^37^^,^^38^ and alternative atlas definitions or parcellations may yield different spatial patterns and effect estimates. Third, the cross-sectional design precludes conclusions about causality or temporal progression. Moreover, because only quantitative [^18^F]FDG-PET data were available in ADNI and only qualitative assessments in MCSA, direct replication was not feasible. Furthermore, both cohorts were predominantly composed of White North American individuals, limiting generalisability. Vascular burden, educational attainment, mixed pathologies, and ethnoracial differences may influence tau-PET patterns, thresholding strategies, and associations with metabolism and cognition.^68^, ^69^, ^70^, ^71^, ^72^, ^73^, ^74^, ^75^ Because SEOT relies on reference-derived thresholds, future studies should evaluate the generalisability of extent-based metrics across more diverse populations and clinical settings. Replication in larger datasets is also warranted. Our relatively small MCSA sample may not be representative of the broader atypical AD population, precluded subgroup analyses, and reduced statistical power to detect differences between tau-PET metrics. Finally, participant selection for the MCSA cohort reflected Canadian clinical practice guidelines^18^ used to determine referrals for [^18^F]FDG-PET, rather than a research-driven sampling strategy. As indications for [^18^F]FDG-PET may differ across healthcare systems and national guidelines, caution is warranted when generalising these findings to settings with different clinical referral practices.

Together, our findings highlight SEOT as a biologically meaningful tau-PET metric that provides information beyond traditional SUVR measures for tracking metabolic and cognitive dysfunction in AD. By capturing a complementary dimension of tau pathology, SEOT may offer additional insights into disease mechanisms, progression, and treatment response. These results support the incorporation of extent-based measures into research, trials, and diagnostic frameworks.

## Contributors

ACM and PRN conceptualised and designed the study. LT, SAH, GB, KQS, JT, NR, CT, MSW, DOL, SM, TC, BH, EA, SS, MPG, PV, KP, TAP, JPS, and PRN contributed to data acquisition and analysis, data interpretation, and revision of the manuscript. CL, LSM, DGS, APBR, JK, and ERZ contributed to data interpretation and revision of the manuscript. ACM drafted the manuscript, and all authors revised it. The ADNI consortium contributed to data acquisition. All authors read and approved the final version of the manuscript. All authors had full access to all the data in the study and had final responsibility for the decision to submit for publication. LT, SAH, GB, KQS, JT, NR, and PRN accessed and verified the data.

## Data sharing statement

Data from the ADNI cohort can be accessed online (https://ida.loni.usc.edu/). All requests for raw and analysed data and materials will be promptly reviewed by McGill University to verify if the request is subject to any intellectual property or confidentiality obligations. Anonymised data will be shared upon request to the study's senior author from a qualified academic investigator for the sole purpose of replicating the procedures and results presented in this article. Any data and materials that can be shared will be released via a material transfer agreement. Data are not publicly available due to information that could compromise the privacy of research participants. Related documents, including study protocol and informed consent forms, can similarly be made available upon request.

## Declaration of interests

ACM received financial support from the Alzheimer Society of Canada Doctoral Award and a travel award to present this work at the Tau 2026 conference by the Alzheimer's Association, the Rainwater Charitable Foundation, and CurePSP. JT has served as a consultant for the Neurotorium Educational Platform and Roche Diagnostics, and as a medical writer for Alzheon Inc, both outside of the scope of the present work. CT received consulting fees from Genentech outside of the scope of this manuscript. MSW receives honoraria from Lilly and Eisai for lectures outside of the scope of this manuscript. LSM received research grants and/or scholarships from the Anna-Lisa och Bror Björnsons Stiftelse, Adlerbert Research Foundation, Gun och Bertil Stohnes Stiftelse, Stiftelsen För Gamla Tjänarinnor, Herbert och Karin Jacobssons Stiftelse, Sahlgrenska Academy, Eva och Oscar Ahréns Stiftelse, and Stiftelsen Wilhelm och Martina Lundgrens Vetenskapsfond, as well as travel support from the Alzheimer's Association. DGS received travel support for attending meetings from the Alzheimer's Association and BrainLat/RedLat. TC served on an advisory board for Eisai. PV received honoraria from Eli Lilly, Eisai, and Novo Nordisk for lectures outside of the scope of this manuscript. KP has served as a consultant for Eli Lilly, Biospective, and Optina Diagnostics outside of the scope of this manuscript. TAP received research grants from the National Institute on Aging (5R01AG073267 and 5R01AG075336), travel support from the Alzheimer's Association, participated in a Data Safety Monitoring Board for the University of Cincinnati College of Medicine, and served on a Johnson & Johnson Diagnostics Advisory Board on biomarkers in Alzheimer's disease. ERZ has served on the scientific advisory board, as a consultant or speaker for Nintx, Novo Nordisk, Biogen, Lilly, Magdalena Biosciences and masima. ERZ is also a co-founder and minority shareholder of masima. Outside the work presented in this paper, PRN provides consultancy services for Roche, Cerveau Radiopharmaceuticals, Lilly, Eisai, Pfizer, and Novo Nordisk. PRN also serves as a clinical trials investigator for Biogen, Novo Nordisk, and Biogen. All other authors declare no competing interests.
