# Supplementary material for: Differential associations of tau extent and load with brain metabolic and cognitive dysfunction in Alzheimer's disease
Source: eBioMedicine. 2026 Aug 29;131:106460. doi: 10.1016/j.ebiom.2026.106460 (PMC13545302; doi:10.1016/j.ebiom.2026.106460)
Supplement: Supplementary file 1 [file mmc1.docx]

**Supplementary Table 1. Demographic, clinical, and biomarker characteristics of the threshold analysis sample for the MCSA dataset**

|  | **Aβ-** **CU (n = 138)** | **Aβ+ CI (n = 105)** |
| --- | --- | --- |
| **Age (years), mean (SD)** | 66.1 (9.7) | 70.2 (7.4)** |
| **Female, n (%)** | 83 (60%) | 59 (56%) |
| **Male, n (%)** | 55 (40%) | 46 (44%) |
| **Years of education, mean (SD)** | 15.7 (3.5) | 14.7 (3.8)* |
| **MMSE, mean (SD)** | 29.2 (0.9) | 24.6 (5.7)**** |
| **Temporal meta-ROI tau-PET SUVR, mean (SD)** | 1.0 (0.1) | 2.3 (1.1)**** |
| **Neocortical Aβ-PET SUVR, mean (SD)** | 1.3 (0.1) | 2.4 (0.5)**** |
| **APOE ε4 carriers, n (%)** | 37 (27%) | 61 (60%)**** |
|  |  |  |

Group differences were assessed using the Wilcoxon rank–sum test for continuous variables (age, education, MMSE, and SUVR measures) and the χ² test for categorical variables (sex and APOE ε4 carriership). Statistically significant differences are displayed when present. AD: Alzheimer’s disease; APOE: apolipoprotein E; CU: cognitively unimpaired; MCI: mild cognitive impairment; MMSE: Mini-Mental State Examination; SD: standard deviation.

*p < 0.01, **p < 0.01, ****p < 0.0001

**Supplementary Figure 1. Discriminatory performance of whole-cortex SEOT across voxel-level thresholds. A** presents a line graph showing the AUC values of cortical SEOT for distinguishing Aβ− cognitively unimpaired individuals from Aβ+ cognitively impaired individuals from the TRIAD cohort across voxel-level thresholds ranging from 1.0 to 3.0 SUVR values. The highest AUC values are indicated with arrows. These analyses were performed to determine the optimal voxel-level threshold subsequently applied to the MCSA subset. No participants included in these threshold analyses were part of the MCSA subset. **B** shows the frequency with which each voxel-level SUVR threshold was selected as the optimal threshold across 1,000 bootstrap resamples of the TRIAD derivation cohort. In each bootstrap iteration, participants were resampled with replacement, ROC analyses were repeated across all candidate thresholds, and the threshold yielding the highest AUC was recorded. Thresholds of 1.6, 1.7, and 1.9 were most frequently selected, supporting the robustness of threshold selection.

**Supplementary Table 2. Demographic characteristics of reference control samples used to derive voxelwise z-scored SEOT measures.**

|  | **TRIAD/MCSA (n=40)** | **ADNI (n=40)** |
| --- | --- | --- |
| **Age (years), mean (SD)** | 59.5 (9.1) | 63.1 (3.7) |
| **Female, n (%)** | 24 (60%) | 21 (52.5%) |
| **Male, n (%)** | 16 (40%) | 19 (47.5%) |
| **Years of education, mean (SD)** | 15.0 (3.0) | 16.1 (2.4) |

All participants were cognitively unimpaired, Aβ− controls with available tau-PET imaging acquired using the same tracer as the corresponding analytical dataset. SD: standard deviation.

**Supplementary Figure 2. Participant flow for the ADNI and MCSA analytical samples.**

**ADNI**

**2,280 available [¹⁸F]flortaucipir tau-PET scans**

↓

364 participants with a corresponding same-visit [¹⁸F]FDG-PET scan
(no repeated participants)

↓

Excluded (n = 214) for the following reasons:
MRI, [¹⁸F]FDG-PET, and [¹⁸F]flortaucipir imaging quality control failure: n = 15
No usable Aβ biomarker data: n = 79
Aβ− status: n = 120

↓

**Final ADNI sample: n = 150**
**6 CU • 97 MCI • 47 dementia due to AD**

**MCSA**

**967 available tau-PET scans**

↓

74 participants with an available [¹⁸F]FDG-PET scan
For each participant, the tau-PET scan closest in time to [¹⁸F]FDG-PET was selected

↓

Excluded (n = 30) for the following reasons:
Aβ− status: n = 30

↓

**Final MCSA sample: n = 44**
**18 MCI • 26 dementia with atypical AD clinical features**

Aβ: amyloid-β; AD: Alzheimer’s disease; ADNI: Alzheimer’s Disease Neuroimaging Initiative; CU: cognitively unimpaired; FDG: fluorodeoxyglucose; MCI: mild cognitive impairment; MCSA: McGill Centre for Studies in Aging; MRI: magnetic resonance imaging; PET: positron emission tomography.

**Supplementary Table 3. Sensitivity analyses including amyloid-β burden as a covariate in linear models assessing associations between tau-PET metrics and [^18^F]FDG-PET meta-ROI SUVR.** Models were run in the respective samples with available data for each amyloid-β biomarker. Standardised β coefficients, 95% confidence intervals, and p-values are shown for the amyloid-β biomarker term, together with ΔBIC values relative to the base model including the tau-PET predictor, age, and sex.

| **Tau-PET predictor** | **Aβ biomarker added** | **Standardised β**  **(Aβ biomarker)** | **P-value** | **ΔBIC vs base model** |
| --- | --- | --- | --- | --- |
| **Temporal SUVR** | Aβ-PET SUVR | 0.03 (−0.17 to 0.24) | 0.75 | +4.49 |
| **Temporal SEOT** | Aβ-PET SUVR | 0.06 (−0.16 to 0.28) | 0.58 | +4.27 |
| **Cortical SUVR** | Aβ-PET SUVR | 0.02 (−0.18 to 0.23) | 0.83 | +4.55 |
| **Cortical SEOT** | Aβ-PET SUVR | 0.06 (−0.15 to 0.27) | 0.58 | +4.28 |
| **Temporal SUVR** | CSF Aβ₄₂/₄₀ | −0.004 (−0.18 to 0.17) | 0.96 | +4.71 |
| **Temporal SEOT** | CSF Aβ₄₂/₄₀ | −0.02 (−0.20 to 0.16) | 0.79 | +4.64 |
| **Cortical SUVR** | CSF Aβ₄₂/₄₀ | 0.02 (−0.15 to 0.20) | 0.78 | +4.63 |
| **Cortical SEOT** | CSF Aβ₄₂/₄₀ | −0.02 (−0.19 to 0.15) | 0.83 | +4.66 |

Aβ: amyloid-β; BIC: Bayesian information criterion; CSF: cerebrospinal fluid; FDG: fluorodeoxyglucose; PET: positron emission tomography; ROI: region of interest; SEOT: spatial extent of tauopathy; SUVR: standardised uptake value ratio.

**Supplementary Table 4. Sensitivity analyses of age- and sex-adjusted associations between tau-PET metrics and [^18^F]FDG-PET meta-ROI signal across different SEOT thresholding approaches.** Standardised β coefficients, 95% confidence intervals (CI), model fit statistics, and Bayesian Information Criterion (BIC) values are shown for separate linear regression models predicting [^18^F]FDG-PET meta-ROI SUVR. SEOT measures were derived using either a fixed global SUVR threshold (1.4) or voxelwise thresholds corresponding to 2, 2.5, and 3 standard deviations above the reference group mean. ΔBIC values are presented relative to the best-performing model (lowest BIC), with lower ΔBIC values indicating stronger model support. Covariates included age and sex.

| **Tau-PET metric** | **Standardised β (CI)** | **p-value** | **Adj. R^2^** | **F-stats** | **BIC** | **ΔBIC vs best model** |
| --- | --- | --- | --- | --- | --- | --- |
| Temporal SUVR | -0.48 \| [-0.64, -0.32] | <0.0001 | 0.21 | 14.41 | -144.13 | 3.68 |
| Cortical SUVR | -0.48 \| [-0.65, -0.32] | <0.0001 | 0.20 | 13.30 | -141.46 | 6.35 |
| Temporal SEOT (1.4 fixed cut-off) | -0.44 \| [-0.60, -0.29] | <0.0001 | 0.19 | 12.74 | -140.11 | 7.70 |
| Cortical SEOT (1.4 fixed cut-off) | -0.51 \| [-0.67, -0.35] | <0.0001 | 0.23 | 15.98 | -147.81 | 0.00 |
| Temporal SEOT (2 SD voxelwise) | -0.39 \| [-0.55, -0.24] | <0.0001 | 0.16 | 10.27 | -133.94 | 13.87 |
| Cortical SEOT (2 SD voxelwise) | -0.46 \| [-0.62, -0.30] | <0.0001 | 0.20 | 13.12 | -141.03 | 6.78 |
| Temporal SEOT (2.5 SD voxelwise) | -0.42 \| [-0.58, -0.27] | <0.0001 | 0.18 | 11.80 | -137.78 | 10.03 |
| Cortical SEOT (2.5 SD voxelwise) | -0.48 \| [-0.64, -0.33] | <0.0001 | 0.21 | 14.56 | -144.48 | 3.33 |
| Temporal SEOT (3 SD voxelwise) | -0.45 \| [-0.60, -0.29] | <0.0001 | 0.19 | 13.03 | -140.81 | 7.00 |
| Cortical SEOT (3 SD voxelwise) | -0.50 \| [-0.66, -0.35] | <0.0001 | 0.23 | 15.64 | -147.02 | 0.79 |

BIC: Bayesian information criterion; CI: confidence interval; FDG: fluorodeoxyglucose; PET: positron emission tomography; ROI: region of interest; SD: standard deviation; SEOT: spatial extent of tauopathy; SUVR: standardised uptake value ratio.

**Supplementary Table 5. Sensitivity analyses of associations between tau-PET metrics and [^18^F]FDG-PET meta-ROI signal across different SEOT thresholding approaches after adjustment for demographic, genetic, and clinical factors.** Standardised β coefficients, 95% confidence intervals (CI), model fit statistics, and Bayesian Information Criterion (BIC) values are shown for separate linear regression models predicting [^18^F]FDG-PET meta-ROI SUVR. SEOT measures were derived using either a fixed global SUVR threshold (1.4) or voxelwise thresholds corresponding to 2, 2.5, and 3 standard deviations above the reference group mean. ΔBIC values are presented relative to the best-performing model (lowest BIC), with lower ΔBIC values indicating stronger model support. All models were adjusted for age, sex, education, APOEε4 carriership, and clinical diagnosis.

| **Tau-PET metric** | **Standardised β (CI)** | **p-value** | **Adj. R^2^** | **F-stats** | **BIC** | **ΔBIC vs best model** |
| --- | --- | --- | --- | --- | --- | --- |
| Temporal SUVR | -0.32 \| [-0.48, -0.16] | <0.0001 | 0.31 | 10.73 | -148.88 | 2.79 |
| Cortical SUVR | -0.31 \| [-0.48, -0.14] | <0.0001 | 0.30 | 10.30 | -146.78 | 4.89 |
| Temporal SEOT (1.4 fixed cut-off) | -0.28 \| [-0.44, -0.13] | <0.0001 | 0.30 | 10.20 | -146.26 | 5.41 |
| Cortical SEOT (1.4 fixed cut-off) | -0.35 \| [-0.51, -0.19] | <0.0001 | 0.33 | 11.31 | -151.67 | 0.00 |
| Temporal SEOT (2 SD voxelwise) | -0.23 \| [-0.39, -0.07] | <0.0001 | 0.28 | 9.35 | - 142.04 | 9.64 |
| Cortical SEOT (2 SD voxelwise) | -0.30 \| [-0.46, -0.14] | <0.0001 | 0.31 | 10.47 | -147.58 | 4.09 |
| Temporal SEOT (2.5 SD voxelwise) | -0.26 \| [-0.42, -0.11] | <0.0001 | 0.29 | 9.85 | -146.59 | 5.08 |
| Cortical SEOT (2.5 SD voxelwise) | -0.32 \| [-0.48, -0.17] | <0.0001 | 0.32 | 10.88 | -151.08 | 0.59 |
| Temporal SEOT (3 SD voxelwise) | -0.29 \| [-0.44, -0.13] | <0.0001 | 0.30 | 10.26 | -144.54 | 7.13 |
| Cortical SEOT (3 SD voxelwise) | -0.34 \| [-0.50, -0.18] | <0.0001 | 0.32 | 11.19 | -149.59 | 2.08 |

BIC: Bayesian information criterion; CI: confidence interval; FDG: fluorodeoxyglucose; PET: positron emission tomography; ROI: region of interest; SD: standard deviation; SEOT: spatial extent of tauopathy; SUVR: standardised uptake value ratio.

**Supplementary Table 6. Sensitivity analyses comparing the accuracy of tau load and tau extent metrics derived using different voxelwise thresholding approaches for identifying [^18^F]FDG-PET hypometabolism.** The table presents AUC values for each tau-PET metric and p-values from pairwise comparisons of AUCs performed using DeLong’s test. SEOT measures were derived using voxelwise thresholds corresponding to 2, 2.5, and 3 standard deviations above the reference group mean. Hypometabolism was defined as an [^18^F]FDG-PET meta-ROI SUVR < 1.21.

| **Tau-PET metric** | **AUC** | **DeLong p vs Temporal SUVR** | **DeLong p vs Cortical SUVR** |
| --- | --- | --- | --- |
| Temporal SUVR | 0.67 | — | — |
| Cortical SUVR | 0.63 | 0.06 | — |
| Temporal SEOT (2 SD voxelwise) | 0.68 | 0.32 | 0.04 |
| Cortical SEOT (2 SD voxelwise) | 0.68 | 0.63 | <0.01 |
| Temporal SEOT (2.5 SD voxelwise) | 0.68 | 0.24 | 0.04 |
| Cortical SEOT (2.5 SD voxelwise) | 0.68 | 0.32 | <0.01 |
| Temporal SEOT (3 SD voxelwise) | 0.69 | 0.17 | 0.03 |
| Cortical SEOT (3 SD voxelwise) | 0.69 | 0.25 | 0.01 |

AUC: area under the curve; FDG: fluorodeoxyglucose; PET: positron emission tomography; ROI: region of interest; SD: standard deviation; SEOT: spatial extent of tauopathy; SUVR: standardised uptake value ratio.

**Supplementary Figure 3. Five-fold cross-validation component tuning curves for the tau SEOT, tau SUVR model, and combined tau SUVR + SEOT machine learning models predicting [^18^F]FDG-PET signatures.** Curves represent the mean cross-validated correlation between predicted and observed [^18^F]FDG-PET distributed regional patterns across increasing numbers of latent partial least squares components. Shaded areas indicate variability across folds. Vertical dashed lines denote the optimal number of latent components selected for the final models. Across all models, predictive performance peaked at five latent components before declining with increasing model complexity, consistent with overfitting at higher component numbers. All models were adjusted for age and sex.

FDG: fluorodeoxyglucose; PET: positron emission tomography; SEOT: spatial extent of tauopathy; SUVR: standardised uptake value ratio.

**Supplementary Table 7. Age- and sex-adjusted associations between network-level [^18^F]FDG-PET SUVR and voxelwise tau-PET extent measures across AD-related networks.** The table displays Spearman’s correlation coefficients between network-level [¹⁸F]FDG-PET SUVR and tau-PET load or SEOT measures derived using voxelwise thresholds of 2, 2.5, and 3 SD above the reference mean. Corresponding p-values are shown in parentheses. Steiger p-values compare each SEOT–FDG correlation against the corresponding tau load–FDG correlation within the same network.

| **Network** | **Tau load vs FDG ρ (p)** | **2 SD SEOT vs FDG ρ (p)** | **Steiger p vs tau load** | **2.5 SD SEOT vs FDG ρ (p)** | **Steiger p vs tau load** | **3 SD SEOT vs FDG ρ (p)** | **Steiger p vs tau load** |
| --- | --- | --- | --- | --- | --- | --- | --- |
| DMN | −0.14 (0.09) | −0.28 (<0.001) | <0.001 | −0.30 (<0.001) | <0.001 | −0.33 (<0.001) | <0.001 |
| FPEC | −0.22 (0.01) | −0.37 (<0.001) | <0.001 | −0.40 (<0.001) | <0.001 | −0.41 (<0.001) | <0.001 |
| Language | −0.42 (<0.001) | −0.49 (<0.001) | 0.05 | −0.51 (<0.001) | <0.01 | −0.52 (<0.001) | <0.01 |
| Salience | −0.03 (0.71) | −0.22 (0.01) | <0.001 | −0.25 (0.003) | <0.001 | −0.25 (0.003) | <0.001 |
| Visuospatial | −0.35 (<0.001) | −0.36 (<0.001) | 0.83 | −0.37 (<0.001) | 0.57 | −0.36 (<0.001) | 0.70 |
| Limbic | −0.36 (<0.001) | −0.38 (<0.001) | 0.42 | −0.39 (<0.001) | 0.18 | −0.40 (<0.001) | 0.14 |

AD: Alzheimer’s disease; FDG: fluorodeoxyglucose; PET: positron emission tomography; SD: standard deviation; SEOT: spatial extent of tauopathy; SUVR: standardised uptake value ratio.

**Supplementary Table 8. Associations between network-level [¹⁸F]FDG-PET SUVR and tau-PET metrics across AD-related networks after adjustment for demographic, genetic, and clinical factors.** The table displays Spearman’s correlation coefficients between network-level [¹⁸F]FDG-PET SUVR and tau-PET load or SEOT measures derived using voxelwise thresholds of 2, 2.5, and 3 SD above the reference mean. Corresponding p-values are shown in parentheses. Steiger p-values compare each SEOT–FDG correlation against the corresponding tau load–FDG correlation within the same network. All correlations were adjusted for age, sex, education, APOEε4 carriership, and clinical diagnosis.

| **Network** | **Tau load vs FDG ρ (p)** | **Fixed 1.4 SEOT vs FDG ρ (p)** | **Steiger p vs tau load** | **2 SD SEOT vs FDG ρ (p)** | **Steiger p vs tau load** | **2.5 SD SEOT vs FDG ρ (p)** | **Steiger p vs tau load** | **3 SD SEOT vs FDG ρ (p)** | **Steiger p vs tau load** |
| --- | --- | --- | --- | --- | --- | --- | --- | --- | --- |
| DMN | −0.04 (0.60) | -0.24 (<0.01) | <0.001 | −0.16 (0.06) | 0.02 | −0.19 (0.02) | 0.001 | −0.22 (<0.01) | <0.001 |
| FPEC | −0.12 (0.16) | -0.31 (<0.001) | <0.001 | −0.26 (<0.01) | <0.001 | −0.29 (<0.001) | <0.001 | −0.30 (<0.001) | <0.001 |
| Language | −0.31 (<0.001) | -0.43 (<0.001) | <0.01 | −0.38 (<0.001) | 0.15 | −0.42 (<0.001) | 0.02 | −0.42 (<0.001) | <0.01 |
| Salience | 0.005 (0.95) | -0.24 (<0.01) | <0.001 | −0.15 (0.08) | <0.001 | −0.20 (0.02) | <0.001 | −0.22 (0.01) | <0.001 |
| Visuospatial | −0.29 (<0.001) | -0.25 (<0.01) | 0.25 | −0.24 (<0.01) | 0.32 | −0.26 (<0.01) | 0.39 | −0.25 (<0.01) | 0.23 |
| Limbic | −0.27 (0.001) | -0.31 (<0.001) | 0.17 | −0.28 (<0.001) | 0.70 | −0.29 (<0.001) | 0.32 | −0.30 (<0.001) | 0.21 |

AD: Alzheimer’s disease; FDG: fluorodeoxyglucose; PET: positron emission tomography; SD: standard deviation; SEOT: spatial extent of tauopathy; SUVR: standardised uptake value ratio.

**Supplementary Figure 4. Voxelwise associations between tau-PET metrics and [^18^F]FDG-PET hypometabolism in competitive models including tau extent and tau load predictors.** The panels show voxelwise t-maps from linear regression models examining the associations between tau-PET metrics and [^18^F]FDG-PET signal while simultaneously accounting for both tau extent (SEOT) and tau load (SUVR) measures within the same model. Each panel is organised according to the competitive model tested (bold titles), with separate maps shown for each predictor retained in the model. Predictors without significant voxelwise associations after correction are not displayed. Covariates included age and sex. Arrowheads indicate small focal clusters to facilitate visualisation. FDG: fluorodeoxyglucose; PET: positron emission tomography; SEOT: spatial extent of tauopathy; SUVR: standardised uptake value ratio.

**Supplementary Table 9. Associations between tau-PET metrics and [^18^F]FDG-PET meta-ROI signal in cognitively unimpaired and mild cognitive impairment participants.** Standardised β coefficients, 95% CI, model fit statistics, and BIC values are shown for separate linear regression models predicting [^18^F]FDG-PET meta-ROI SUVR. SEOT measures were derived using either a fixed global SUVR threshold (1.4) or voxelwise thresholds corresponding to 2, 2.5, and 3 standard deviations above the reference group mean. ΔBIC values are presented relative to the best-performing model (lowest BIC), with lower ΔBIC values indicating stronger model support. All models were adjusted for age and sex.

| **Tau-PET metric** | **Standardised β (CI)** | **p-value** | **Adj. R^2^** | **F-stats** | **BIC** | **ΔBIC vs best model** |
| --- | --- | --- | --- | --- | --- | --- |
| Temporal SUVR | -0.39 \| [-0.59, -0.20] | <0.001 | 0.14 | 6.68 | -105.67 | 4.70 |
| Cortical SUVR | -0.35 \| [-0.55, -0.15] | <0.001 | 0.11 | 5.10 | -101.48 | 8.88 |
| Temporal SEOT (1.4 fixed cut-off) | -0.43 \| [-0.62, -0.24] | <0.0001 | 0.18 | 8.26 | -109.71 | 0.65 |
| Cortical SEOT (1.4 fixed cut-off) | -0.39 \| [-0.58, -0.20] | <0.0001 | 0.14 | 6.74 | -105.85 | 4.52 |
| Temporal SEOT (2 SD voxelwise) | -0.36 \| [-0.55, -0.17] | <0.001 | 0.13 | 5.90 | -103.63 | 6.73 |
| Cortical SEOT (2 SD voxelwise) | -0.35 \| [-0.54, -0.16] | <0.001 | 0.12 | 5.50 | -102.58 | 7.79 |
| Temporal SEOT (2.5 SD voxelwise) | -0.41 \| [-0.59, -0.22] | <0.0001 | 0.16 | 7.39 | -107.52 | 2.85 |
| Cortical SEOT (2.5 SD voxelwise) | -0.37 \| [-0.56, -0.18] | <0.001 | 0.13 | 6.09 | -104.13 | 6.24 |
| Temporal SEOT (3 SD voxelwise) | -0.44 \| [-0.62, -0.25] | <0.0001 | 0.18 | 8.53 | -110.37 | 0.00 |
| Cortical SEOT (3 SD voxelwise) | -0.38 \| [-0.58, -0.19] | <0.001 | 0.14 | 6.51 | -105.23 | 5.13 |

BIC: Bayesian information criterion; CI: confidence interval; FDG: fluorodeoxyglucose; PET: positron emission tomography; ROI: region of interest; SD: standard deviation; SEOT: spatial extent of tauopathy; SUVR: standardised uptake value ratio.

**Supplementary Table 10. Associations between tau-PET metrics and [^18^F]FDG-PET meta-ROI signal in participants with dementia.** Standardised β coefficients, 95% CI, model fit statistics, and BIC values are shown for separate linear regression models predicting [^18^F]FDG-PET meta-ROI SUVR. SEOT measures were derived using either a fixed global SUVR threshold (1.4) or voxelwise thresholds corresponding to 2, 2.5, and 3 standard deviations above the reference group mean. ΔBIC values are presented relative to the best-performing model (lowest BIC), with lower ΔBIC values indicating stronger model support. All models were adjusted for age and sex.

| **Tau-PET metric** | **Standardised β (CI)** | **p-value** | **Adj. R^2^** | **F-stats** | **BIC** | **ΔBIC vs best model** |
| --- | --- | --- | --- | --- | --- | --- |
| Temporal SUVR | -0.19 \| [-0.53, 0.14] | 0.25 | 0.19 | 4.59 | -48.73 | 1.30 |
| Cortical SUVR | -0.23 \| [-0.59, 0.13] | 0.21 | 0.19 | 4.71 | -49.03 | 1.00 |
| Temporal SEOT (1.4 fixed cut-off) | -0.03 \| [-0.34, 0.28] | 0.84 | 0.16 | 4.04 | -47.34 | 2.69 |
| Cortical SEOT (1.4 fixed cut-off) | -0.29 \| [-0.64, 0.07] | 0.12 | 0.21 | 5.12 | -50.03 | 0.00 |
| Temporal SEOT (2 SD voxelwise) | -0.01 \| [-0.32, 0.30] | 0.96 | 0.16 | 4.02 | -47.30 | 2.73 |
| Cortical SEOT (2 SD voxelwise) | -0.26 \| [-0.60, 0.09] | 0.14 | 0.21 | 4.98 | -49.69 | 0.34 |
| Temporal SEOT (2.5 SD voxelwise) | -0.02 \| [-0.33, 0.29] | 0.92 | 0.16 | 4.02 | -47.31 | 2.72 |
| Cortical SEOT (2.5 SD voxelwise) | -0.27 \| [-0.63, 0.08] | 0.13 | 0.21 | 5.04 | -49.85 | 0.18 |
| Temporal SEOT (3 SD voxelwise) | -0.03 \| [-0.34, 0.29] | 0.86 | 0.17 | 4.03 | -47.33 | 2.70 |
| Cortical SEOT (3 SD voxelwise) | -0.28 \| [-0.64, 0.08] | 0.12 | 0.21 | 5.09 | -49.97 | 0.06 |

BIC: Bayesian information criterion; CI: confidence interval; FDG: fluorodeoxyglucose; PET: positron emission tomography; ROI: region of interest; SD: standard deviation; SEOT: spatial extent of tauopathy; SUVR: standardised uptake value ratio.

**Supplementary Figure 5. Stage-stratified voxelwise associations between tau-PET metrics and [^18^F]FDG-PET hypometabolism.** Panel **A** shows t-maps from voxelwise linear regressions between tau-PET metrics (predictors in separate models) and [^18^F]FDG-PET SUVR (outcome), adjusted for age and sex in pre-dementia participants (i.e., cognitively unimpaired and MCI participants). Panel **B** shows analogous voxelwise associations in participants with dementia. Tau-PET predictors are indicated above each brain map. FDG: fluorodeoxyglucose; MCI: mild cognitive impairment; PET: positron emission tomography; SEOT: spatial extent of tauopathy; SUVR: standardised uptake value ratio.
